# Next-generation nanomedicine for cancer therapy: advances, challenges, and future perspectives

**DOI:** 10.3389/fchem.2026.1920337

**Published:** 2026-09-17

**Authors:** Biswajit Mohanty, Puja Saikia, Piyush Pandey, Chira R. Bhattacharjee

**Affiliations:** 1 Department of Chemistry, Assam University, Silchar, Assam, India; 2 Departement of Microbiology, Assam University, Silchar, Assam, India

**Keywords:** cancer therapy, nanocarriers, nanomedicine, next-generation nanovaccine, targeted drug delivery

## Abstract

In recent times, permeation of nanotechnology to modern medicine has revolutionized transformation, especially in the case of cancer diagnosis and therapy, positioning nanomedicine as a key pillar of next-generation healthcare. Tuning the physicochemical properties of nanoparticles such as size, morphology, and surface charge, enables controlled targeted drug delivery, increased cellular uptake and controlled therapeutic release. Nanomedicine-based platforms can improve certain drawbacks associated with conventional therapies including poor aqueous solubility, unfavorable pharmacokinetics, systemic toxicity and low bioavailability for specific therapeutic agents. While consistent tumor-specific accumulation, intratumoral heterogeneity, drug resistance, and long-term therapeutic outcomes remains challenging and highly formulation- and patient-dependent. This review paper besides, focussing on individual nanocarrier classes or specific therapeutic modalities, also provide an integrated and critical perspective on next-generation nanomedicine by connecting nanocarrier design with tumor-specific accumulation, intracellular trafficking, stimuli-responsive drug release, combination therapy and clinical translation. Recent advances in polymeric, lipid-based, inorganic, biological, and hybrid nanocarriers are systematically discussed, with particular stress on surface engineering, ligand-mediated targeting, multifunctional delivery systems and nanoengineered cancer medicine. The potential of these platforms for elevating therapeutic precision, overcoming biological barriers, multidrug resistance and enabling site-specific delivery is critically studied. In addition, major translational challenges, including large-scale fabrication, reproducibility, long-term safety, regulatory barriers, and nanotoxicological concerns are also addressed. Finally, emerging directions involving personalized nanomedicine, combination therapies, and artificial intelligence-assisted nanoparticle design are highlighted to provide perspectives toward safer, more effective, and patient-concentrated cancer therapy.

## Graphical Abstract

**Figure:**
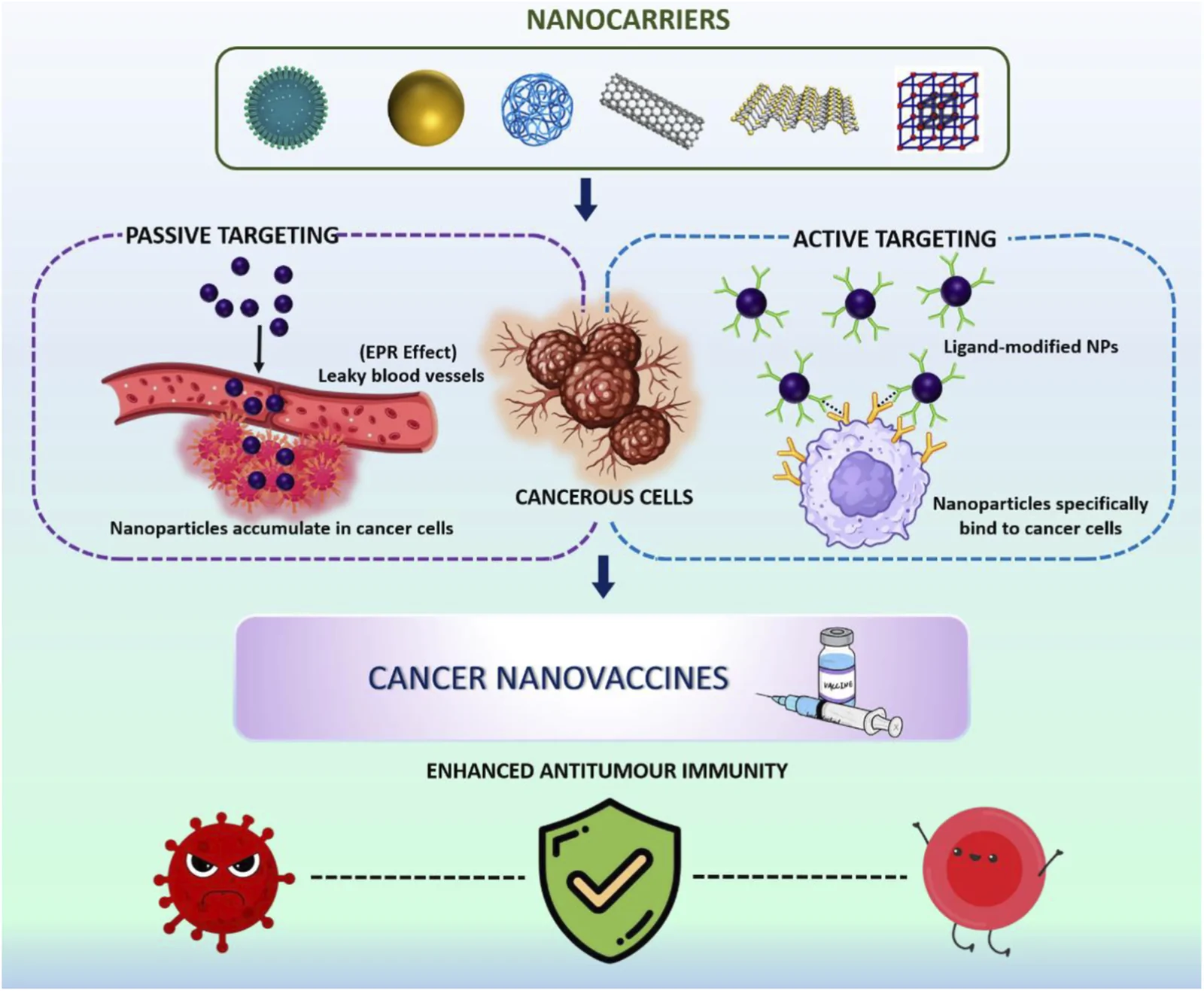

## 1 Introduction

Over the past couple of decades, remarkable advancements have been accomplished in relation to the development of a new class of engineered materials called “nanomaterials.” The field has rapidly expanded influencing almost every sector, including academia, industries and everyday public applications. This emerging area of research is driven by the engineered materials at the nanoscale (typically less than 100 nm in at least one dimension), its physicochemical properties can differ significantly from those of its bulk counterpart. Such nanoscale materials often exhibit enhanced conductivity, mechanical strength, high surface-area-to-volume ratio, and unique optical properties which can be effectively exploited for diverse technological and scientific applications. Among other fields influenced by nanomaterials, pharmacology and medicine remains one of the most promising and impactful areas. Nanomaterials possess the potential to considerably improve healthcare sector in terms of nanosized engineered medicine that can emerge as novel therapeutic agents. By the development of this nanomedicine, the efficacy, targeted delivery, biocompatibility and therapeutic performance can be substantially enhanced (Mishra et al., 2010; Neubert, 2011). Furthermore, nanoscale drug delivery systems can minimize off-target interactions, thereby reducing toxicity and side effects. Therefore, nanomedicines have opened new avenues for the treatment of various diseases, including cancer treating nanomedicine, a rapidly advancing frontier in modern medicine (Thorley and Tetley, 2013).

A systematic literature search was conducted in the Web of science database, covering a 10-year period (2016–2026), with the final search performed on 22 May 2026. The search strategy was based on the keywords nanomedicine, nanovaccine, cancer, and cancer therapy, using the Boolean operators AND and OR. Only English-language research articles and review articles published during the specified period were considered. Editorials, conference papers/proceedings, book chapters, letters, notes, and other non-research document types were excluded. Records unrelated to cancer-focused nanomedicine/nanovaccine applications were also excluded. Duplicate records were identified and removed before screening. Landmark studies were prioritized based on their scientific significance and citation impact, whereas recent studies were included to capture emerging developments and current research trends. Bibliographic data were exported from Scopus and analyzed using VOSviewer for keyword co-occurrence, country-wise contribution, subject-area distribution, and publication trends (Figure 1).

**FIGURE 1:**
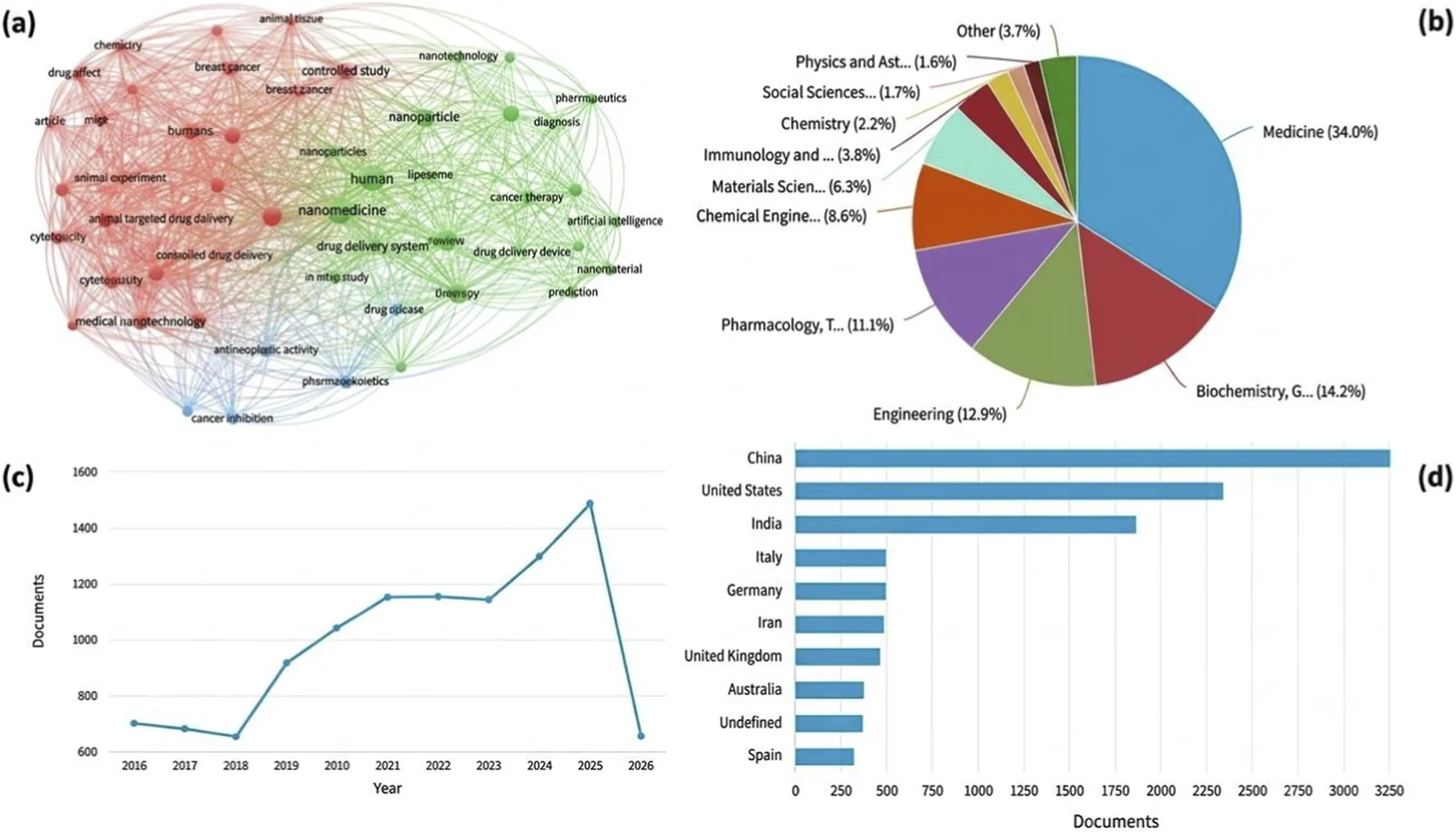
**(a)** Global publication distribution depicted on a world map **(b)** Publication by field **(c)** Publication number of next-generation cancer vaccine in last 10 years **(d)** Publication by country.

Cancer has emerged as one of the deadliest global health challenges acknowledged as leading cause of mortality worldwide. As per the reports published by World Health Organization 2020, approximately 10 million cancer related deaths were reported globally, with the most casualty occurring in low to middle-income countries. The most prevalent forms of cancer are breast, lung, colorectal, prostate, skin and gastric all of which poses significant challenges to healthcare systems worldwide (Sung et al., 2021). Chemotherapy continues to be the most commonly employed cancer treatment alongside surgery and radiotherapy due to its broad applicability, economic, and therapeutic effectiveness. Notwithstanding potent cytotoxic activity and improved patient survival rates, several challenges remain. The cause of non-selective nature of chemotherapeutic agents often results in significant off-target toxicity, causing adverse effects in healthy tissues and thereby, reducing patient tolerability and diminished the quality of life. Frequently reported side effects include peripheral neuropathy, nausea, fatigue, myelosuppression, alopecia, nephrotoxicity, and cardiotoxicity (Debela et al., 2021). Furthermore, poor aqueous solubility of many anticancer drugs hinder the formulation of drugs contributing to unfavorable pharmacokinetic behavior, including low bioavailability and inadequate therapeutic concentrations at targeted tumor site. Another challenges that frequently emerges is the adapting resistance to drug by the cancer cells that ultimately diminishes the effectiveness of chemotherapy (Weber, 2014). New developments in cancer therapeutics have introduced several promising treatment strategies - stem targeted therapy, cell therapy, gene therapy, ablation therapy, and natural antioxidant-based therapies each with distinct advantages and limitations (Debela et al., 2021).

Given the limitations of conventional chemotherapy, development of efficient drug delivery systems has become a key focus in cancer research. Inspite of notable advances in cancer therapeutics, achieving optimal drug concentration at the tumor site with minimum systemic toxicity remains a significant challenge. The therapeutic performance of conventional anticancer agents is compromised by poor solubility, rapid degradation, low bioavailability, short circulation half-life, and inadequate tumor selectivity. Hence, balancing the biological fate of active pharmaceutical ingredients (APIs) after administration has become a critical feature of modern drug development (Wu et al., 2011). Nanocarriers possesses advantages by encapsulating a wide range of therapeutic molecules, thereby protecting them from enzymatic degradation and premature clearance while increasing their solubility and stability in biological atmospheres. Such encapsulation not only helps in improving the pharmacokinetic and pharmacodynamic properties of drugs but also allows controlled, sustained, and stimuli-responsive release profiles. Moreover, advances in nanoparticle engineering have facilitated the development of sophisticated delivery platforms incorporating passive targeting, ligand-mediated active targeting, and externally triggered release mechanisms (Desai, 2012). An important feature of nanocarriers lies in theircapability to favorably accumulate within tumor tissues through the increased permeability and retention (EPR) effect. This effect comes from the leaky vasculature and impaired lymphatic drainage typical of the tumor microenvironment (TME). This phenomenon enhances intra-tumoral drug retention and endorses sustained therapeutic action and thereby increasing treatment efficacy and reduced damage to healthy tissues (Ngoune et al., 2016).

A wide variety of nanomaterials have been reported for cancer-targeted therapy, including polymeric nanoparticles, liposomes, dendrimers, inorganic nanomaterials, polymeric micelles, hydrogels, and carbon-based nanostructures (Baher, 2009; Prabhu et al., 2014; Riaz et al., 2016; Sun et al., 2021). Recent studies dealt with the development of multifunctional nanoparticles system that are capable of delivering multiple therapeutic agents simultaneously. Combination therapy and dual-drug delivery strategies are of rising concerndue to their synergistic drug interactions, improve therapeutic outcomes, and help overcome multidrug resistance. Co-delivery of drugs by nanomaterials further provide drug protection, facilitate sustained and controlled drug release and improving the drug mechanism of action at the target site. Thus, by dual targeting strategyof drugsenables selective accumulation in cancer cells while simultaneously directing therapeutic agents to specific subcellular organelles like mitochondria, nuclei and the endoplasmic reticulum (Fu et al., 2020). More than 200 FDA approved protein- and peptide-based therapeutics have been approved for the treatment of various diseases, but their clinical efficacy is often restricted due to poor membrane permeability, immunogenicity, short plasma half-life. Encapsulation of these proteins/peptides within nanoparticles or surface conjugation strategies can protect these biomolecules from degradation, prolong circulation time, and enhance cellular uptake. Through hydrophobic ion pairing technique, this encapsulation can be done more effectively (Ristroph and Prud’homme, 2019; Malik and Bahal, 2019).

The clinical translation of nanomaterial-based therapeutic drugs has been progressed substantially over the past 2 decades. Several nanoformulations have got the regulatory approval or advanced into clinical trials, underlining their growing significance in cancer treatment. Notably, nanoparticle-mediated delivery systems have successfully facilitated the clinical administration of small interfering RNA (siRNA) and other nucleic acid therapeutics for the treatment of solid tumors (Henn et al., 2024). The clinical success of nanomedicine was first demonstrated by the approval of the liposomal formulation of doxorubicin, DOXIL®, which marked a significant milestone in the field of cancer therapy (Barenholz, 2012). Since then, nanomedicine has evolved substantially, leading to the development of diverse therapeutic and diagnostic agents. Nanocarrier technologies in cancer therapy have gained prominence in other biomedical fields, particularly vaccine delivery, as evidenced by their critical role in several SARS-CoV-2 vaccine formulations (Aldosari et al., 2021; Khurana et al., 2021). Nanomedicine has also invaded to the field of photodynamic therapy (PDT) for cancer treatment which is an invasive cancer treatment that use a photosensitizer, light, and oxygen to produce reactive oxygen species (ROS). The clinical efficacy of conventional photosensitizers is often limited by poor solubility, low ROS generation, and inadequate tumor targeting which can be addressed by use of nanomedicine. Kumar and Abrahamse (2021) studied the application of various biocompatible nanocarriers in PDT and reported encouraging results in both *in vitro* and *in vivo* studies. Furthermore, Li and Kataoka (2021) also reported the development of next-generation nanomedicine platforms for targeted cancer therapy.

Although numerous reviews have addressed nanomedicine-assisted cancer therapy, the rapidly evolving scenario of next-generation platforms needs a broader and more integrated study. The emerging dimensions of cancer nanomedicine, such as nanoengineered cancer vaccines, personalized therapeutic agent, and artificial intelligence-assisted nanoparticle design, are developing quite fast and need consideration within a framework. Therefore, there is a requirementof a comprehensive review that not only summarizes recent nanomedicine platforms but also critically connects their structural and functional design with therapeutic performance, safety, translational feasibility, and future clinical applicability. This review summarizes recent advances in next-generation nanomedicine for cancer therapy, focusing on innovative nanocarrier systems, nanoengineered cancer vaccines, and targeted delivery strategies. The roles of metallic, carbon-based, silica-based, lipid-based, polymeric, and biological nanoplatforms in improving therapeutic efficacy and precision are discussed. Additionally, the mechanisms of passive and active targeting, along with current challenges and future prospects for the clinical translation of cancer nanomedicines are critically evaluated.

## 2 Mechanism of cellular targeting

Targeted drug delivery is an effective strategy in cancer therapy that enables the selective accumulation of therapeutic agents at tumor sites while minimizing toxicity to other healthy cells. The physicochemical characteristics of nanocarriers and the characteristics of the tumor microatmosphere influence the targeting mechanism. To improve therapeutic outcomes and tide over treatment challenges, two key targeting strategies: passive targeting and active targeting have been utilizedto enhance the precise drugs and genes delivery to cancer cells.

### 2.1 Passive targeting

Passive targeting is one of the earliest and most commonly employed strategies in nanomedicine-based cancer therapy (Figure 2A). The targeting mechanism is based on the enhanced permeability and retention (EPR) effect first reported by Hiroshi Matsumura and Yasuhiro Maeda in 1986 (Matsumura and Maeda, 1986), where nanocarriers preferentially gather in specific tumor tissues due to the unique abnormal vascular and lymphatic characteristics of the tumor environment (Hansen et al., 2015). Though these abnormalities associated with tumor facilitate nanocarrier accumulation, the extent and mechanism of nanoparticle entry vary with vascular architecture, tumour type and nanoparticle properties. It has also been proved that active endothelial transport, in addition to passive passage through vascular defects, can also contribute to nanoparticle entry into solid tumours (Torchilin, 2011).

**FIGURE 2:**
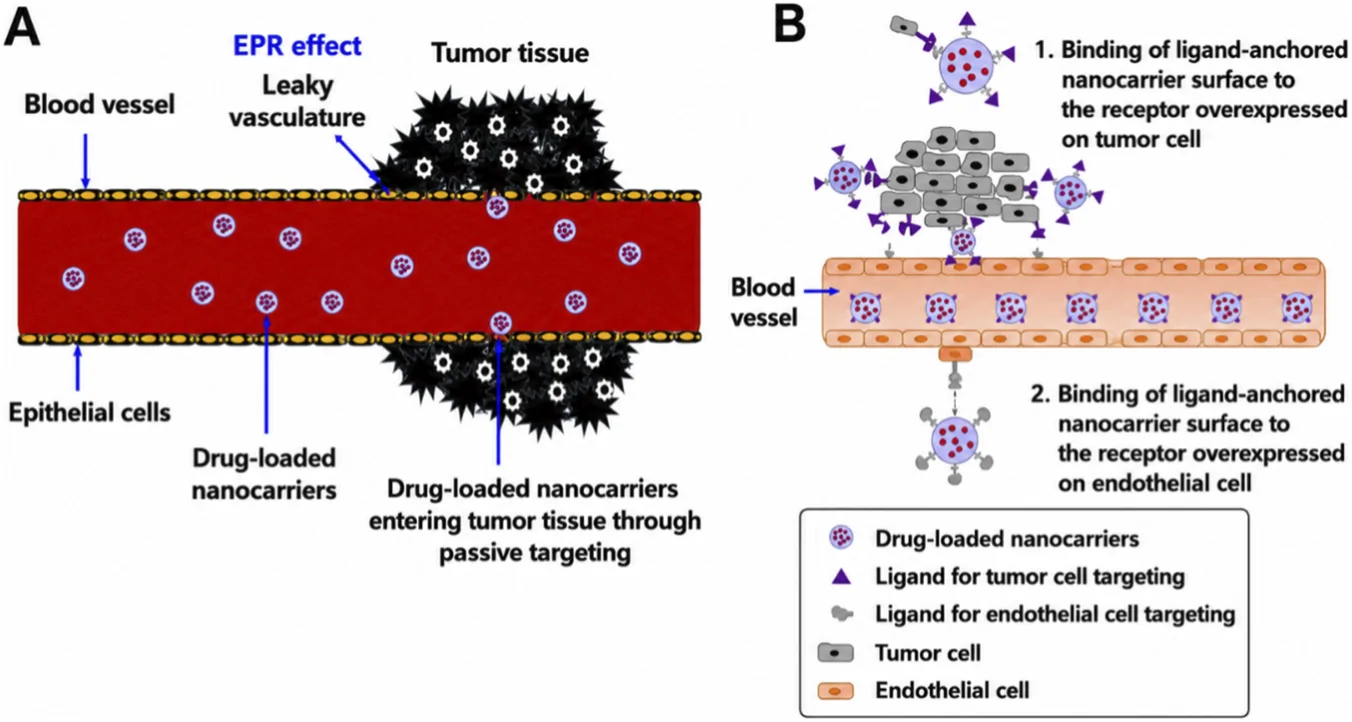
Representation of **(A)** passive targeting **(B)** active targeting mechanism by nanocarriers (adapted from Din et al., 2017).

The effectiveness of passive targeting is greatly influenced by the physicochemical properties of nanocarriers, including particle size, surface charge, and circulation time in terms of vascular transport, tumour accumulation, interstitial penetration and clearance. However, the optimum values are context dependent and difficult to generalize based on tumor types or nanocarrier platofrms. Surface modifications such as using polyethylene glycol (PEG) can increase the circulation half-life, reduce opsonization and improve tumor accumulation. Besides vascular abnormalities, passive targeting also exploits distinctive features of the tumor microenvironment (Tsujimoto et al., 2021). Tumors typically possess hypoxia, acidic pH, elevated metabolic activity and the excessive expression of enzymes such as matrix metalloproteinases. These characteristics have encouraged the development of stimuli-responsive nanocarriers capable of releasing therapeutic agents selectively within the tumor tissues. Hence, tumour delivery is considered as a multistep process rather than a single accumulation event, comprising at least five interconnected stages: (i) vascular transport and extravasation, (ii) interstitial transport and intratumoral penetration, (iii) cellular uptake, (iv) intracellular trafficking and drug release, and (v) pharmacodynamic action at the intended molecular target. Numerous nanocarrier platforms, including liposomes, dendrimers, polymeric nanoparticles, micelles, hydrogels and antibody-based systems have been reported to enhance passive tumor targeting and drug retention (Attia et al., 2019). Several clinically tested and approved nanomedicines are used via passive targeting mechanisms, including liposomal formulations such as Doxil, DaunoXome, Marqibo, Onivyde, and albumin-bound nanoparticles such as Abraxane. These formulations have showed improved therapeutic ability and reduced harmful effects by exploiting prolonged systemic circulation and EPR-mediated tumor accumulation (Barai, 2026; Shi et al., 2017).

Despite of the listed advantages, passive targeting faces significant limitations due totumor heterogeneity and interpatient variability. Factors such as elevated interstitial fluid pressure, dense stromal barriers and poor vascular perfusion and hypopermeable tumor tissues can severely hinder the nanoparticle penetration into the tumor cores. Experimental examination indicate that tumour-associated macrophage and vascular density can serve as predictors of nanomedicine accumulation, further supporting the importance of tumour-specific biological features in determining delivery efficiency. Along with this, sequestration by the liver and spleen often limits the fraction of administered nanoparticles reaching the target site. In many cases, only a small fractionof the administered dose accumulates the tumor site, with drug distribution mostly confined to well-vascularized peripheral regions (Torchilin, 2011).

Recent studies emphasised improving transportation of nanoparticle and intra-tumoral distribution. Strategies such as size-transformable nanocarriers, vascular normalization using anti-angiogenic agents, tumor-responsive delivery systems and microenvironment-modulating therapies have shown promise in improving this targeting challenges The limitations associated with passive targeting leads to the development of active targeting strategies that combine EPR-mediated accumulation with receptor-specific cellular uptake (Subhan et al., 2021).

### 2.2 Active targeting

In active targeting approach, nanocarriers are chemically modified with specific ligands that recognize receptors or molecular markers expressed on tumour cells, tumour vasculature, stromal components or immune cells within the tumour microenvironment (TME) (Figure 2B). Active targeting strategy is based on the Paul Ehrlich’s early 20th-century “magic bullet” concept that selectively deliver therapeutic agents to targeted tissues while minimizing damage to healthy ones (Strebhardt and Ullrich, 2008). Unlike passive targeting, active targeting usesspecific ligands such as monoclonal antibodies, antibody fragments (scFv), peptides (e.g., RGD), transferrin, folic acid, aptamers, growth factors and nucleic acids to target disease cells rather than using the EPR effect. The nanocarriers with conjugated ligands target receptors that are highly expressed on cancer cells or tumor-associated endothelial cells (Golombek et al., 2018). However, ligand conjugation does not by itself ensure selective tumour delivery, since targeting efficiency depends on many factors like receptor abundance, receptor accessibility and heterogeneity, ligand affinity and orientation, ligand density, and the rate and mechanism of receptor internalization. Experimental studies have demonstrated that nanoparticle uptake depends on both ligand density and receptor expression, with an optimal ligand–receptor matching being required for efficient receptor-mediated endocytosis. Ligand-receptor interactions generate receptor-mediated endocytosis, increasing intracellular delivery of therapeutic agents, such as proteins, siRNA and nucleic acid therapeutics. Though, receptor binding and cellular internalization do not necessarily result in productive intracellular delivery because internalized nanocarriers may undergo endosomal sequestration or lysosomal trafficking rather than release their cargo at the intended intracellular site. Furthermore, the formation of a protein corona in biological fluids can mask surface ligands and reduce their accessibility to receptors, thereby altering targeting performance. (Chamundeeswari et al., 2019).

Active targeting delivery can be targeted to either cancer cells or their vasculature. Tumor-cell targeting generally uses receptors like the folate receptor (FR), transferrin receptor (TfR), epidermal growth factor receptor (EGFR), human epidermal growth factor receptor 2 (HER2) and prostate-specific membrane antigen (PSMA) (Deshpande et al., 2013). Nanocarriers functionalized with folate have shown potential against FR-positive breast, lung, ovarian and colorectal cancers, while that are modified with transferrin systems have been utilized for delivering drug across the blood-brain barrier. However, the therapeutic benefit of tumour-cell targeting depends on sufficient receptor expression and accessibility throughout the tumour, and heterogeneous receptor expression can produce spatially variable nanoparticle uptake. As an alternative, vascular targeting approach focuses on markers such as vascular endothelial growth factors (VEGFs), matrix metalloproteinases (MMPs), vascular cell adhesion molecules (VCAMs), and αvβ3 integrins which disrupt the tumor blood supply and inhibiting growth (Danhier et al., 2010). That’s why, vascular targeting is distinguished from tumour-cell targeting because the targeted compartment, receptor accessibility and intended therapeutic mechanism are different. Targeting tumour-associated endothelial cells may facilitate vascular disruption or alter the tumour vasculature, whereas targeting tumour cells aims primarily to enhance cellular uptake and intracellular drug delivery.

Several studies have discussed the advantages of active targeting such as transferrin-functionalized docetaxel nanocrystals showed an improved uptake and cytotoxicity in A549 lung cancer cells, while cyclic RGD-modified apatinib-loaded liposomes exhibited an increased uptake and tumor inhibition in HCT116 colon cancer cells through αvβ3 integrin targeting (Song et al., 2017). Similarly, folate-conjugated paclitaxel-loaded polymeric micelles and actively targeted paclitaxel-functionalized polymeric micelle nanofibers displayed superior antitumor efficacy compared with non-targeted formulations (Wu et al., 2012). These findings demonstrate the potential of ligand-mediated cellular recognition, but improved cellular uptake in receptor-positive experimental models should not be equated with universal improvement in tumour biodistribution or penetration. Indeed, primary studies have shown that targeting ligands can increase intracellular localization without necessarily increasing total tumour accumulation.

In comparison with passive targeting systems, actively targeted nanocarriers can achieve higher intracellular drug delivery and enhanced tumor selectivity. However, active targeting does not necessarily increase the total amount of nanocarrier reaching the tumour. Rather, its principal contribution may occur after vascular delivery by increasing the fraction of tumour-associated nanoparticles that interact with receptor-positive cells. Systematic studies of ligand density demonstrated that changes in ligand density can alter pharmacokinetics and biodistribution, indicating that functionalization itself can modify systemic disposition (Bazak et al., 2014). Antibody-conjugated HER2-targeted nanoparticles have proven enhanced uptake and therapeutic efficacy in HER2-positive breast cancer models. Functionalization with dual ligands, such as RGD/transferrin co-functionalized nanocarriers, has been investigated to combine recognition of multiple cellular or vascular targets and potentially enhance receptor-mediated uptake. The benefit of multiligand functionalization depends on receptor co-expression, ligand accessibility, affinity and the effects of surface modification on biodistribution (Dahiya et al., 2021).

The clinical possibility of active targeting is reflected in several nanomedicines investigated for receptor-mediated delivery, including MM-302 (HER2-targeted liposomal doxorubicin), BIND-014 (PSMA-targeted docetaxel nanoparticles), MBP-426 (TfR-targeted liposomal oxaliplatin) and anti-EGFR immunoliposomes carrying doxorubicin. The clinical success of antibody–drug conjugates (ADCs), including Adcetris, Kadcyla, Sacituzumab Govitecan and Trastuzumab deruxtecan, further demonstrates that receptor recognition can be therapeutically exploited when the target is sufficiently expressed and accessible and when internalization, intracellular processing and payload release are appropriately coupled. However, the success of ADCs should not be interpreted as evidence that ligand-functionalized nanoparticles inherently achieve selective tumour biodistribution, because these platforms differ substantially in size, pharmacokinetics, payload properties and mechanisms of tissue distribution (Amiri-Kordestani et al., 2014).

Active targeting needs to be treated as a downstream step within the overall nanomedicine delivery system rather than as an independent mechanism of tumour localization. Following systemic administration, a ligand-functionalized nanocarrier first maintain proper circulation, reach the tumour-associated vasculature, extravasate or otherwise access the relevant tumour compartment, and penetrate the tumour microenvironment before ligand–receptor interactions can occur. Subsequently, receptor-specific functionalization cannot fully compensate for poor biodistribution, inadequate tumour circulation or limited intratumoral penetration. Experimental studies proved that targeting ligands can significantly increase nanoparticle localization within receptor-positive tumour cells without necessarily increasing the total amount of nanoparticle accumulated in the tumour. Thus, the outcome of active targeting should be evaluated at multiple levels, including tumour accumulation, spatial penetration, cellular uptake, intracellular trafficking and therapeutic response, rather than solely by measuring total tumour-associated nanoparticle content.

Active targeting is also associated with drawbacks such as heterogeneous receptor expression, ligand shedding, immune-mediated clearance and endosomal entrapment. To address these limitations, strategies have been developed such as pH-responsive fusogenic peptides, stimuli-responsive linkers, and MMP-sensitive ligand-masking systems. Overall, active targeting increases the accuracy, effectiveness and safety of cancer nanomedicine by merging selective ligand–receptor interactions with the drug-carrying capacity of nanocarriers and hence improving therapeutic outcomes withminimum systemic toxicity (Kachore and Chambhare, 2024).

## 3 Types of nanocarriers (advances in next-generation nanocarriers)

Colloidal particles less than 500 nm, known as nanocarriers, are utilized for the encapsulation of biological materials and drugs. They can be composed of different materials, which comprise (i) inorganic nanocarriersthat includes metal and metal oxide nanoparticles (like gold, silver, and iron oxide), silica-based structures such as mesoporous silica nanoparticles, carbon-based materials including graphene and carbon nanotubes (Jin et al., 2009), and semiconductor quantum dots. Each of these has distinct physicochemical characteristics, such as magnetic responsiveness or photothermal conversion. (ii) Organic nanocarriers consist of lipid-based systems (such as liposomes, nanostructured lipid carriers, solid lipid nanoparticles and nanoemulsions) (Kreilgaard, 2001), polymer-based platforms (including polymeric nanoparticles, micelles, dendrimers, nanogels, and polymersomes) (Pushp et al., 2012), and vectors derived from biomolecules like protein nanoparticles, peptide assemblies (Morachis et al., 2012), nucleic-acid nanostructures, and exosomes. (iii) Hybrid nanocarriers incorporate two or more types of materials-such as lipid-polymer hybrids, cell-membrane-coated biomimetic systems, organic-inorganic composites—aimed at merging structural strength with biological selectivity (Nimeshet, 2012). This idealclassification clarifies the comparison of nanocarrier properties, mechanisms, and therapeutic applications in precision oncology (Figure 3).

**FIGURE 3:**
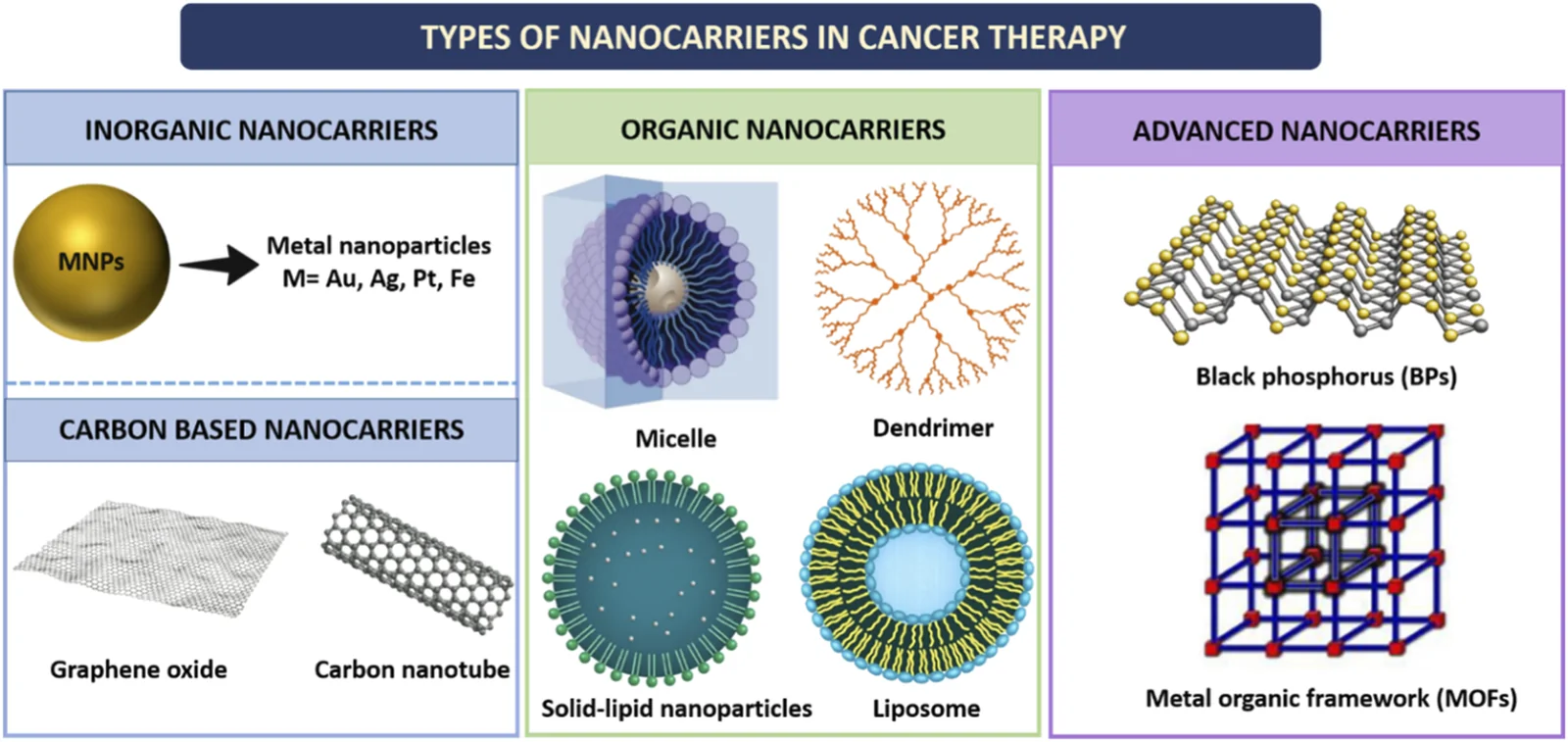
Representation of various types of nanaocarriers in cancer therapy.

### 3.1 Inorganic nanocarriers

Inorganic nanocarriers consist of materials such as metal (e.g., silver and gold), metal oxides (e.g., titanium oxide, iron oxide, zinc oxide, and copper oxide), mesoporous silica, GO, carbon nanotubes, and black phosphorous (Majhi and Yadav, 2021; Pugazhendhi et al., 2018; Shi et al., 2020). These nanocarriers can be in the form of nanoshells, nanocages, nanorods, nanostars, and nanospheres. Inorganic nanoparticles are primarily advantageous due to their elevated stability in comparison with organic materials (Paul and Sharma, 2010). Such materials can address instability problems, like susceptibility to oxidation and hydrolysis that lipid-meadiated nanocarriers face. Furthermore, drugs can readily leak from lipid-based nanocarriers, especially inorganic ones, show promise as an alternative for drug delivery (Pugazhendhi et al., 2018). In addition to their diverse surface chemistry and controllable structures, other notable benefits of inorganic nanocarriers include unique magnetic, electrical, and optical properties (Kashapov et al., 2021; Liu et al., 2021). In addition to their potential for drug delivery, several inorganic nanoparticles are utilized in PDT, PTT, and hyperthermia therapy (Yang et al., 2020). While they provide a lot of prospective benefits, their biocompatibility and biodegradability are generally low. Various inorganic nanocarriers have been extensively studied for cancer diagnostic and treatment purposes, including iron nanoparticles (FeNPs), mesoporous silica nanoparticles (MSNs), gold nanoparticles (AuNPs), carbon nanotubes (CNTs) and GO for treatment purposes.

#### 3.1.1 Metal nanoparticles

Owing to the exclusive physicochemical properties, metal NPs received immense attraction particularly with regard to drug delivery, cancer diagnosis and treatment, (Paresishvili and Kakabadze, 2023). The NPs can be formed with a controllable size and shape, and to coat their surface with stealth ligands so that they can evade the immune system and remain in longer blood circulation (Sharma et al., 2022). Metal NPs viz., Ag, Au, Ti,Pd, Zn and Cu NPs, demonstrate improved optical properties and can be modified to link targeting agents and active biomolecules through covalent or electrostatic interactions like hydrogen bonds (Chandrakala et al., 2022). Due to large surface-to-volume ratio, they can be chemically modified, taken up more readily by cells, used to shield drugs from the biological medium, and made more bioavailable (Desai et al., 2021).

##### 3.1.1.1 Gold nanoparticles

Gold nanoparticles have emerged as promising cancer therapy agents for various applications, including drug carriers, photothermal agents, radiosensitizers and contrast agents (Hossain et al., 2024; Lee et al., 2017). They can be simply linked to biomolecules via Au-S bonds, enhancing their usefulness in biomedical applications (Rosero et al., 2024). Metal-based nanoparticles, especially those made of gold and silver, due to their distinctive optical characteristics may be utilized for successful photothermal therapy. For instance, gold nanoparticles can absorb near-infrared light and produce heat, which can result in the destruction of localized tumours while reducing harm to adjacent healthy tissues. By allowing the targeting of drugs to cancer cells while protecting normal tissues, current progress in nanoparticle manufacturing has greatly improved cancer treatment. Stimulus-responsive nanoparticles enable controlled drug discharge, confirming targeted action against tumours (Pawar et al., 2023). AuNPs are utilized in gene therapy and photothermal treatment (PTT) for the shift of nucleic acids into tumour cells, representing a potential new frontier in molecular medicine for cancer therapy (Huang et al., 2024). This approach removes cancer cells and reduces damage to healthy tissues thereby improving treatment efficacy. While DOX is a very effective chemotherapy drug, it has considerable toxic effects on organs such as the kidneys, heart and liver (Fu et al., 2023). The *in vivo* and in activity of DOX-loaded DNA/Au NPs for ovarian cancer treatment has been investigated (Lee et al., 2020). They studied the discharge of DOX from the DOX-DNA-Au NPs in variouspH buffers. An primary burst release of DOX was seen within 5 h, with higher release under acidic conditions, indicating pH-responsive drug delivery. The DOX-DNA-Au NPs showed high stability, efficient cellular uptake, and successful nuclear localization in ovarian cancer cells. *In vivo* studies demonstrated significantly enhanced tumor inhibition rate 2.5 times compared to free DOX, with minimal accumulation in other organs, suggesting effective and targeted anticancer activity with reduced side effects. Multifunctional transition metal carbonitride MXenes (Ti_3_C_2_ MXene-Au) nanocomposites that combines photothermal therapy, enzyme-assisted therapy, and antitumor immunotherapy within a single system. In addition, the platform demonstrated dual-mode photoacoustic and thermal imaging capabilities *in vivo*, highlighting its potential for advanced cancer diagnosis and therapeutic applications (Chang et al., 2022). A pH, enzyme and NIR responsive immune-adjuvant nanoparticle system (RMmAGL) was synthesized for tumour-specific photothermal and immunotherapeutic applications. It was designed to release functionalized AuNPs and the Toll like receptor 7 (TLR7) agonist R837 in acidic tumor surrounding enabling enzyme-activated photothermal therapy under NIR irradiation with activation of dendritic cell and T-cell. Further, mannose-functionalized silica nanoparticles encouraged macrophage polarization toward an antitumor phenotype, causingsubstantial suppression of primary tumours and metastasis in both *in vitro* and *in vivo* studies (Liu et al., 2023). The potential of gold nanoparticle/PLA polymer nanospheres as an effective theranostic platform has been demonstrated with AuNP > 50 nm exhibiting strong near-infrared (NIR) responsiveness for efficient therapeutic performance, whereas ultrasmall 4 nm gold nanoparticles incorporated within polymeric nanoclusters enabled enhanced renal clearance, thus improving biocompatibility and reducing long-term accumulation (Tam et al., 2010). Despite significant advances in gold nanoparticle-based theranostic systems, a comprehensive understanding of the influence of nanoparticle size and shapeon organ-specific biodistribution remains limited. Moreover, the development of long-lived luminescent gold nanoparticles (L-AuNPs) with enhanced stability and imaging efficiency presents a promising research direction for advanced biomedical application.

##### 3.1.1.2 Silver nanoparticles

Inorganic nanoparticles, particularly silver nanoparticles (AgNPs), have gained increasing attention owing to their wide-ranging applications in areas such as catalysis (Rahimkhoei et al., 2020) and biomedicine (da Silva et al., 2021; El-Rafie et al., 2013; Razavi et al., 2021). They have been extensively explored as nanocarriers owing to their excellent conductivity, high chemical stability, and promising therapeutic potential (Gounden et al., 2021). Silver nanoparticles exhibiting strong gene-binding affinity have emerged as promising gene delivery agent for the treatment of different diseases, including cancer (Sharma et al., 2022). Recently, a chitosan (CHI)-activated cisplatin-loaded silver nanoparticles composite system was fabricated showedrequired properties, including small particle size, high positive zeta potential and an efficient drug delivery capacity greater than 80% for cisplatin. Notably, even without the incorporation of a specific targeting moiety, the system showed minimal cytotoxicity toward non-cancerous cells while demonstrating strong selectivity towards breast cancer cells, achieving more than 80% cell death at low drug dosages (Gounden et al., 2021). Researchers have examined the effects of selenic acid (SA)- and pyruvic acid (PA)-loaded silver nanocarriers on the feasibility of colorectal cancer (CRC) cells. The synthesized AgNPs were sizes ranging from 2.32 to 5.61 nm, while Ag-SA and Ag-PA nanoparticles showed size distributions of 3.61–13.54 nm and 78.82–295.3 nm, respectively. A continuous growth in the collective release of SA and PA from the nanoparticles was studied over time. Biological evaluation displayed that Ag-SA NPs significantly reduced CRC cell viability, whereas Ag-PA NPs revealeduseless anticancer activity. Furthermore, PA alone was non-cytotoxic and promoted CRC cell propagation, while SA enhanced the cytotoxic effect of AgNPs by approximately 5.3fold, inducing clear apoptotic and necrotic cell death in treated cancer cells (Erdemir et al., 2024). Chitosan-capped Silver nanoparticles (Ag NPs-CHI) demonstrated strong and selective anticancer property against breast cancer cells while exhibiting minimal toxicity toward healthy human cells. The nanocomposite effectively inhibited the expression of inflammatory markers like IL-6 and TNF-α, unlike conventional AgNO_3_, which showed lower selectivity and higher toxicity toward healthy cells. Owing to their stability, scalability, cost-effectiveness, and reduced side effects, these nanoparticles show considerable promising as a potential therapeutic platform for breast cancer treatment (Abdellatif et al., 2023).

##### 3.1.1.3 Platinum nanoparticles

Cisplatin and other platinum-based chemotherapeutic agents, including carboplatin, oxaliplatin, and nedaplatin have long been employed as standard treatments for various cancers. However, though clinically effective, these drugs are frequently associated with severe toxicity and significant adverse side effects limiting their therapeutic applications (Kang et al., 2015). In a bid to overcome these issues, platinum-based nanoparticles were explored owing to their capacity to preferably accumulate at tumour region and increased cellular uptake by cancer cells. This targeted delivery method improves therapeutic efficiency while diminishing systemic toxicity and related side effects (Cheng and Liu, 2017). Novel platinum (IV)-based prodrugs have been modified to reduce off-target relation and minimize toxicity toward healthy cells (Gibson, 2019). These prodrugs undergo intracellular activation within cancer cells, leading to the controlled release of cytotoxic platinum (II) species for effective anticancer therapy (Ferrari et al., 2021). An injectable and degradable photothermal hydrogel based on platinum NPs functionalized with dendrimer (DEPts) and modified dextran was developed for recurring photothermal therapy (PTT). The hydrogel showed exceptional photothermal performance, prolonged tumour retention, and efficient tumour regression while maintaining good biocompatibility. Importantly, the imine bond-crosslinked hydrogel gradually degraded after treatment, thus minimizing the long-term toxicity associated with persistent photothermal nanoparticles and indicating significant potential for benign and efficient cancer therapy (Li et al., 2018). Researchers have studied injectable thermo-sensitive hydrogel system based on copolymer micelles was developed for the localized co-delivery of Gemcitabine and Cisplatin in pancreatic cancer treatment. The dual-drug-loaded hydrogel exhibited reversible sol–gel transition behaviour, sustained drug release, enhanced apoptotic activity, and synergistic inhibition of pancreatic cancer cell proliferation. *In vivo* studies demonstrated superior antitumour activity and minimized systemic toxicity compared to free drugs and single-drug formulations, highlighting the hydrogel’s potential as an effective platform for combination chemotherapy in solid tumors (Shi et al., 2019). Recently, a multicomponent two-layer fibrin-based gel (MCPFTG) was developed for simultaneous tumor suppression and wound healing in squamous cell carcinoma. The dual-layer gel incorporated cisplatin-loaded collagen microcarriers along with bone marrow stem cell-derived factors to enhance tissue regeneration. *In vivo* studies demonstrated significant inhibition of tumor recurrence, reduced cisplatin-associated toxicity, and accelerated wound healing showing the potential of this multifunctional platform for effective postoperative cancer therapy (Kakabadze et al., 2022).

##### 3.1.1.4 Iron nanoparticles

In recent years, iron nanoparticles (FeNPs) have attracted extensive interest for their applications in diagnostics, therapeutics, and theranostics. The nanoparticles can be designed using various chemical, physical, and biological techniques; among all chemical procedures, especially the coprecipitation technique, is the most frequently used technique owing to its ease synthesis and cost-effectiveness (Dadfar et al., 2019; Ngema et al., 2021; Sodipo and Aziz, 2016). The superparamagnetic nature of iron oxide nanoparticles enables their extensive use as contrast agents in magnetic resonance imaging (MRI) and magnetic particle imaging (MPI) (Dulińska-Litewka et al., 2019). The clinical applications of Iron nanoparticles (FeNPs) including iron enrichment, magnetic hyperthermia, macrophage polarization, and magnetically targeted drug delivery (Dadfar et al., 2019). However, their magnetic properties are highly dependent on factors such as composition and morphology. Consequently, careful selection of the preparation method is essential to obtain FeNPs with appropriate shape, size, and crystallinity for optimized biomedical performance. These materials are quite promising in the context of drug delivery applications, magnetic nanoparticles present certain limitations, including difficulties in maintaining therapeutic efficacy at the target site after removal of the external magnetic field. Furthermore, achieving efficient and uniform intratumoral distribution under *in vivo* conditions remains challenging because of the complex three-dimensional architecture of tumor tissues (Schneider-Futschik and Reyes-Ortega, 2021). Doxorubicin is a potent topoisomerase II inhibitor with broad-spectrum anticancer activity; however, its therapeutic efficacy against glioblastoma multiforme (GBM) is significantly restricted by poor penetration across the blood–brain barrier. To overcome this limitation, doxorubicin-loaded, trimethoxysilylpropyl-ethylenediamine triacetic acid (EDT)-stabilized biocompatible iron nanoparticles (FeNPs) were developed to improve targeted delivery and enhance therapeutic effectiveness against GBM (Norouzi et al., 2020). A multifunctional mesoporous silica-coated iron nanoparticles (FeNPs) system, DOX@MMSN-SS-PEI-cit, was developed for MRI-guided targeted cancer therapy. The nanoplatform exhibited pH- and redox-responsive behavior, enabling enhanced cellular uptake and rapid intracellular release of doxorubicin in the tumor microenvironment. *In vitro* and *in vivo* studies demonstrated improved tumor-targeted drug delivery, efficient MRI imaging capability, and superior tumor growth inhibition compared to free doxorubicin (Wan et al., 2020).

#### 3.1.2 Carbon basednanomaterials

Research on carbon-based nanomaterials, including Carbon nanotubes (CNTs), fullerenes, graphene, nanodiamonds, and carbon nanoparticles, has expanded rapidly in due to their significant potential as transport platforms for therapeutic drugs and imaging currently agents in biomedical applications (Thomas et al., 2022; Wen et al., 2021). These carbon-based nanomaterials possess distinctive physicochemical properties, such as high surface-to-volume ratio, robust structural stability, excellent thermal conductivity, and favorable biological compatibility, making them highly suitable for a wide variety of biomedical utilization. Accordingly, these Carbon-based nanomaterials can serve as efficient carriers for hydrophobic drugs, antibodies, antigens, and nucleic acids targeting cancer cells. Moreover, their capability to interact with targeting molecules and aromatic drugs through hydrophobic interactions and supramolecular π–π stacking improves drug-incorporation efficiency and enables sustained and controlled drug release, thereby improving their performance as advanced drug delivery platforms (Sajjadi et al., 2021). Functionalization of carbon-based NMs can be done through non-covalent or covalent bond to improve their biological-compatibility and facilitate integration with various therapeutic and diagnostic agents. In addition, their adjustable pore structure enables controlled and sustained drug release, making them highly effective for advanced biomedical and drug delivery applications (Thomas et al., 2022).

##### 3.1.2.1 Carbon nanotube

Carbon nanotubes (CNTs) are nanoscale porous tubular structures composed of carbon atoms, first discovered by Sumio Iijima in 1991 (Iijima, 1991). CNTs are fullerene-based carbon allotropes consisting of seamless cylindrical graphene sheets and are broadly classified into: multi-walled carbon nanotubes (MWCNTS) and single-walled carbon nanotubes (SWCNTs) and (Liu et al., 2011). Typically, SWCNTs possess outer diameters in the range of 0.4–2 nm, whereasMWCNTs exhibit comparatively larger diameters ranging from 2 to 100 nm (Madani et al., 2011). Mostly employed techniques for the synthesis of carbon nanotubes (CNTs) include laser ablation, chemical vapor deposition (CVD) and arc discharge, including both thermal and plasma-enhanced CVD techniques (Yan et al., 2007). Single-walled carbon nanotubes (SWCNTs) are generally considered more efficient drug delivery agent than multi-walled carbon nanotubes (MWCNTs) due to their well-defined structure and more structural imperfections (Eatemadi et al., 2014). Furthermore, their exceptional thermodynamic and optical properties have made them highly promising candidates for applications in cancer sensing, bioimaging, and therapeutic (Sun et al., 2023). CNTs can be modified via covalent or non-covalent approaches to enhance their biomedical applicability (Li et al., 2017). Functionalization, particularly PEGylation, plays a vital role in improving aqueous dispersibility, prolonging systemic circulation, minimizing recognition by the reticuloendothelial system, and reducing cytotoxicity (Lay et al., 2011). For instance, cyclosporin A (CsA) has been conjugated to amine-terminated phospholipid–PEG-functionalized SWCNTs via a breakable ester linkage, demonstrating the capability of PEGylated SWCNTs as effective drug delivery platforms (Hadidi et al., 2013). Recent investigations have further revealed that appropriately functionalized CNTs have potential of traversing the blood–brain barrier, expanding their applicability in central nervous system therapeutics (Wang and Al-jamal, 2015). In addition to drug delivery, CNTs have shown considerable promise as carriers for diverse genetic materials, including plasmid DNA, antisense oligonucleotides, small interfering RNA (siRNA), and aptamers (Yang et al., 2006). Their unique physicochemical properties also enable their use in cancer therapy through photothermal ablation of tumour tissues (Bao et al., 2018). Furthermore, functionalized CNTs have surfaced as promising diagnostic nanoplatforms for the preliminary detection and surveillance of cancer (Chen et al., 2018).

##### 3.1.2.2 Graphene oxide

Graphene serves as a fundamental building block of various graphitic carbon materials, forming 0D spherical structures (fullerenes), 1D tubular structures (carbon nanotubes, CNTs), and 3D layered structures (graphite) (Geim, 2009; Itoo et al., 2022). It consists of a planar hexagonal lattice of *sp* ^*2*^-hybridized carbon atoms. Owing to its efficient drug-incorporation potential, excellent biological-compatibility, tunable amphiphilicity, and adjustable shape and size graphene and its derivatives are widely explored for cancer-related applications such as biosensing, drug transport, and photothermal therapy (Alemi et al., 2020). Additionally, graphene possesses a large surface-area, outstanding electrical and thermal conductivity, a high strength-to-weight ratio, and remarkable mechanical strength (Papageorgiou et al., 2017; Yadav et al., 2022).

Graphene oxide (GO), an oxidized derivative of graphene, is highly attractive for biomedical utilization due to its facile synthesis, enhanced stability, improved aqueous dispersibility, and favorable optical properties. As a result, numerous GO-based nanomaterials have been developed by integrating GO or its modified forms with chemotherapeutic agents, antibodies, and other nanomaterials (Priyadarsini et al., 2018; Zhou and Bongiorno, 2013). For instances, multifunctional and biocompatible GO–Fe_2_O_4_ conjugates were designed for dual MRI/fluorescence imaging and magnetic targeting (Gonzalez-Rodriguez et al., 2019). These conjugates exhibited a mean diameter of 260 nm and enabled high doxorubicin loading through noncovalent interactions. The conjugates were successfully taken up by cancer cells and demonstrated 2.5-fold higher efficacy than free doxorubicin, achieving comparable cytotoxicity at only one-eighth of the drug dose.

### 3.2 Organic nanocarrier

#### 3.2.1 Liposomes

Liposomes are phospholipid-based spherical vesicles widely employed in drug delivery, particularly for cancer therapy. They offer numerous benefits, including the ability to encapsulate both hydrophilic and hydrophobic drugs, low toxicity, controlled drug release, high loading capacity, and protection of therapeutic agents from external degradation. Their biocompatibility, biodegradability, and low immunogenicity make them attractive drug delivery systems (DDS). However, liposomes also face limitations such as potential carrier-related toxicity, stability issues, drug leakage, and difficulties in large-scale manufacturing (Gatto et al., 2024; Hamad et al., 2024; Kumbham et al., 2023). Liposomal drug delivery has emerged as a highly successful strategy in cancer therapy. Several formulations, including Vyxeos®, Onivyde®, Marqibo®, DepoCyt®, and Doxil®, have been approved for clinical use and commercialized for cancer therapy. These formulations have demonstrated significant potential in enhancing drug delivery while modifying the pharmacokinetic and toxicity profiles of the encapsulated drugs (Kumbham et al., 2023).

Beyond single-drug delivery, liposomes also provide an attractive platform for co-delivery of multiple therapeutic agents within the same vesicle. Co-encapsulation can facilitate the simultaneous exposure of tumour cells to two agents and may improve their spatial co-localization. For example, liposomal co-delivery of β-carotene and doxorubicin has recently been investigated for colorectal cancer, demonstrating the potential of combining a chemotherapeutic drug with a complementary bioactive compound in a single nanocarrier (Alhazzaa et al., 2025). However, co-encapsulation does not necessarily ensure that both drugs remain available at the same ratio throughout storage, circulation, and tumour accumulation. Differences in drug–lipid interactions and membrane partitioning may result in different release rates and consequently alter the drug ratio over time. Moreover, encapsulation of a drug within liposomes does not completely eliminate the possibility of systemic or organ toxicity associated with the therapeutic agent. Liposomes have also been investigated for therepurposing of drugs with unfavourable pharmacokinetic or cellular-delivery properties. PEGylated liposomal metformin, for example, has recently been investigated for hepatocellular carcinoma, providing an example of how nanoformulation may improve the cellular activity and delivery characteristics of an established drug (Elzanaty et al., 2025). However, improved *in-vitro* cellular activity does not by itself demonstrate enhanced tumour accumulation or clinical benefit. Such conclusions require supporting evidence from biodistribution, pharmacokinetic, normal-hepatocyte and organ-toxicity studies, as well as repeated-dose *in-vivo* experiments. Importantly, a correction to this study was subsequently published and should be considered when using quantitative data or figures from the original report. The therapeutic potential of multifunctional liposomes loaded with indocyanine green (ICG), tirapazamine (TPZ), and Ce6 was assessed in A549 tumor-bearing nude mice. Time-dependent fluorescence imaging confirmed tumour targeting and accumulation of the liposomes. *In vivo* PDT activation and immunofluorescence studies revealed that laser irradiation induced pronounced hypoxia through continuous oxygen consumption, thereby activating TPZ and enabling tumour-selective combination therapy (Dai et al., 2019). Hydrophobic hydroxyurea (HU)-mediated nanoliposomes were developed for breast cancer treatment. The HU-loaded nanoliposomes exhibited an average size of 174 nm, comparable to that of the blank, and showed an initial rapid drug release over 5 h followed by sustained release for up to 36 h. This biphasic release behaviour was attributed to surface-associated HU, liposomal erosion, and diffusion-mediated drug transport. Furthermore, the nanoliposomes showed efficient cellular internalization and uptake by breast cancer cells, resulting in enhanced intracellular accumulation of HU (Akbari et al., 2020). Recent developments include thermosensitive liposomes that release drugs upon temperature variations, thereby improving therapeutic targeting while reducing adverse effects (Regenold et al., 2023). The use of liposomes in pediatric cancer therapy is still restricted; however, improvements in liposome design hold promise for modifying drug delivery in pediatric cancer treatment (Feng et al., 2022). Dual pH-responsive liposomes represent an innovative approach for site-specific drug release in tumours, and their use alongside immunotherapy shows potential for enhanced antitumour responses (Zhai et al., 2021). The combination of liposomal DNA transfer and DNA barcoding provides an efficient platform for evaluating and predicting treatment responses. Importantly, liposomes should not be considered merely as inert containers for therapeutic molecules. The encapsulated payload can interact with the lipid bilayer and alter membrane packing, thickness, fluidity, curvature, stiffness, permeability, and leakage behaviour. Recent combined molecular-dynamics (MD) simulations and atomic force microscopy (AFM) studies of doxorubicin-associated BSA-cushioned DPPC bilayers have provided a multiscale perspective linking drug–protein–lipid interactions with measurable changes in membrane properties (Elabed and Abbase, 2026). Such studies demonstrate how molecular-level interactions can influence the mechanical and structural behaviour of lipid membranes and potentially affect drug retention and release. However, these findings should be interpreted cautiously because simulated bilayers are simpler than circulating liposomes, BSA does not reproduce the complexity of the plasma protein corona, and membrane-level changes alone do not establish therapeutic performance. Therefore, serum stability, protein-corona profiling, drug leakage, biodistribution, and *in-vivo* pharmacokinetic studies remain necessary to establish the translational relevance of these biophysical observations.

Overall, liposomes represent one of the most clinically advanced and versatile nanocarrier platforms for drug delivery. Their phospholipid architecture enables the delivery of both hydrophilic and hydrophobic payloads, while surface modification and lipid composition can be adjusted to regulate circulation, cellular uptake, tumour accumulation, and drug release. They are particularly suitable for drugs requiring improved solubility, altered pharmacokinetics, controlled release, or combination delivery. However, tumour accumulation remains dependent on particle size, surface properties, tumour vascularization and tumour heterogeneity, and should not be attributed solely to the enhanced permeability and retention (EPR) effect. Similarly, stimulus-responsive release does not guarantee selective drug release *in vivo* because premature leakage, variable tumour microenvironments, and differences in release kinetics can influence therapeutic performance. Although liposomal formulations generally exhibit favourable biocompatibility, toxicity may still arise from the encapsulated drug, lipid composition, formulation impurities, immune interactions, or long-term exposure. Reproducible control of particle size, lipid composition, encapsulation efficiency, drug-to-lipid ratio, release kinetics, sterility, and storage stability also remains essential for large-scale manufacturing. Thus, the major strength of liposomes lies in their clinical maturity, formulation versatility, and ability to modify the pharmacokinetics and delivery of established drugs, whereas their major challenges include reproducible manufacturing, long-term stability, control of drug release and co-encapsulated drug ratios, tumour-specific biodistribution, and demonstration of clinically meaningful benefit beyond improved *in-vitro* cytotoxicity.

Beyond photothermal and photodynamic approaches, liposomal systems have also been explored as potential radiosensitizers for enhancing the response of tumour cells to ionising radiation. PEGylated β-carotene-loaded liposomes have been investigated in HepG2 cells using *in-vitro* and computational approaches, suggesting a potential interaction between β-carotene nanoformulation and radiation-mediated anticancer effects (Abdel-Moamen et al., 2026). However, these findings should be considered preliminary, as cytotoxicity assays alone cannot establish radiosensitisation. Demonstration of a genuine radiosensitising effect requires radiobiological validation through clonogenic survival assays, determination of dose-enhancement factors, and assessment of radiation-induced DNA damage and repair kinetics using markers such as γH2AX, together with studies under hypoxic and three-dimensional tumour conditions, clinically relevant fractionated radiation schedules, and appropriate normal-tissue toxicity models. The β-Carotene-loaded nanoliposomes have demonstrated anticancer activity against PC-3 prostate cancer cells, including effects on cellular viability, apoptosis and cell-cycle behaviour (Khedr et al., 2026). However, such findings demonstrate anticancer activity rather than receptor-mediated tumour targeting. Molecular targeting requires a defined ligand–receptor pair, evidence of receptor expression, selective ligand binding, blocking or competition experiments, enhanced internalisation relative to an appropriate untargeted control, and preferably selective tumour biodistribution *in vivo*. Therefore, tumour-cell cytotoxicity or cellular uptake alone should not be interpreted as evidence of active targeting.

#### 3.2.2 Solid lipid NPs

Solid lipid nanoparticles (SLNs) are colloidal drug delivery approachthat were first established in the early 1990s, having size range ∼50–1,000 nm. They comprise of a solid lipid core supported by a surfactant layer in watermedium, where the lipid stays in solid form at room and body temperature. The solid matrix helps in smooth drug incorporation, controlled and stable delivery of drug by providing a lipophilic environment (Malam et al., 2009; Müller et al., 2000). Usually SLNs are formed by dispersing melted solid lipids into an aqueous surfactant phase, generally following high-pressure homogenization and microemulsification methods (Barai et al., 2019). Drugs can be incorporated in different structural orientation varying on lipid composition (drug, lipid, surfactant) and processing conditions (hot or cold homogenization, together with a homogeneous matrix, drug-enhanced shell, or drug-loaded core model. These configurations markedly influence drug release behavior and loading capacity (Müller et al., 2002). A variety of lipids are used in SLN formulation, including triglycerides (tristearin, tripalmitin, trimyristin, trilaurin), partial glycerides (glyceryl behenate, glyceryl palmitostearate, glyceryl stearate), fatty acids (stearic acid, capric acid, palmitic acid), steroids (cholesterol) and waxes. Common surfactants contain poloxamers, lecithin, polysorbates, sodium glycocholate, sorbitan esters and their mixtures. Identification of appropriate lipid and surfactant systems is critical, as it defines the particle size, surface charge, stability, drug loading and release mechanism (Barai et al., 2021; Naseri et al., 2015). SLNs have synergistic advantages of polymeric nanoparticles, liposomes and nanoemulsions. Their vital facility contains solvent-free or less toxic production, scalability, easy sterilization, high drug carrying capacity, inhibition of drug degradation, increased bioavailability of weakly water-soluble drugs, controlled release and low cytotoxicity (Mehnert and Mader, 2001). In cancer treatment, SLNs are particularly helpful for lipophilic chemotherapeutic agents such as docetaxel, doxorubicin, paclitaxel, methotrexate and 5-fluorouracil. Advanced engineering, including polymer–lipid hybrid nanoparticles and lipid–drug conjugate systems, have further extended their ability to deliver both lipophilic and hydrophilic or ionic drugs (ud Din et al., 2017).

Several reports highlights their enhanced anticancer efficacy. For example, SLNs combined with linalool (synthesized using cetyl esters, myristyl myristate, cetyl palmitate or with Pluronic® F68) exhibited size in the nano range (∼90–130 nm), thin size distribution, near-neutral zeta potential (∼4 mV), and encapsulation efficiency above 80%. It also showed higher cytotoxic behavior against HepG2 and A549 cancer cells (Rodenak-Kladniew et al., 2017). Similarly, curcumin-functionalized SLNs exhibited higher cytotoxicity against SKBR3 breast cancer cells thanbare curcumin (Wang et al., 2018). Targeted SLN systems have also been synthesized for enhanced tumor specificity. An RGD peptide-functionalized, pH-sensitive doxorubicin-loaded SLN validated a particle size of ∼96 nm, increased encapsulation efficacy (∼98.5%) and pH-sensitive biphasic release, showing elevated drug release in acidic tumor environments. It showed expressively higher bioavailability (5.58-fold increased AUC) and superior inhibition of tumor cellsthan bare free doxorubicin and non-targeted SLNs (Zheng et al., 2019). Despite having these advantages, SLNs are bound with several limitation like rapid clearance by the reticuloendothelial system (RES), problems in encapsulating hydrophilic or ionic drugs and difficulty in precisely controlling drug release profiles.

#### 3.2.3 Micelle

Micelles or polymeric micelles (PMs) are basically colloidal carriers having size in the nanometer range (typically 10–100 nm) that are generated via self-organization of amphiphilic, di-, tri-, or block copolymers in water environments. PMs declare it’s potentially as nanocarriers in cancer therapy bearing unique core-shell structure, especially for the transportation of weakly water-soluble anticancer medicine. Their structure comprises of a hydrophobic core surrounded by a hydrophilic shell (corona). The role of hydrophobic core is the reservoir for encapsulating insoluble therapeutic agents, while the hydrophilic shell stabilizes the micelle, enhances aqueous dispersibility, extends blood circulation and impacts*in vivo* pharmacokinetics (Zhu and Liao, 2015). Micelle formation occurs naturally when amphiphilic copolymers are present above a particular concentration known as the critical micelle concentration (CMC) which also plays a crucial role in decisive micellar stability and assembly. However, micelles may dissociate at some point below the CMC, causing premature drug release (Biswas et al., 2016). PMs are very much suitable for passive targeting of tumor through the increased permeability and retention (EPR) effect owing the smaller size. Usually hydrophilic shell composed of non-reactive polymers like polyethylene glycol (PEG) that helps in minimizing protein adsorption and recognition by the reticuloendothelial system (RES). It enables prolonged circulation and protected encapsulated drugs from degradation within the biological environment (Haider et al., 2022). Apart from size and structure, PMs owns many favorable properties, including biocompatibility, low toxicity, high drug-loading ability, structural flexibility, controlled drug release, and excellent stability that make it potential to use in cancer therapy. Through surface modification of PMs with tumor-oriented ligands or stimuli-responsive systems, it can be useful for active tumor targeting also, that respond to changes in pH, temperature, or other tumor-associated conditions. Some examples of micelle-forming polymers are PEGylated polylactic acid (PEG-PLA), PEGylated polyglutamic acid, PEGylated polyaspartic acid (PEG-PAA), PEGylated poly (lactic-co-glycolic acid) (PEG-PLGA) and PEGylated polycaprolactone (PEG-PCL). Drugs encapsulated PMs can be prepared through physical encapsulation or chemical conjugation, while common preparation methods include dialysis, oil-in-water emulsion, co-solvent evaporation, solvent evaporation and freeze-drying techniques (Biswas et al., 2016).

Various anticancer drugs containing methotrexate, cisplatin, docetaxel, paclitaxel and doxorubicin have been successfully encapsulated in PMs and several micellar formulations are currently undergoing clinical evaluation. One such example is a paclitaxel-loaded micellar formulation (Genexol-PM) that has been accepted for the treatment of non-small cell lung cancer (NSCLC), metastatic breast cancer and ovarian cancer (Keam et al., 2019; Lamey et al., 2023 reported sodium caseinate micelles co-loaded with dasatinib (DAS) and celecoxib (CXB) for breast cancer treatment. These dual drug-loaded micelles showed sustained drug release, with releasing of CXB quicker than DAS due to differences in hydrophobicity and compatibility with the micellar core. The formulation showed minimal hemolytic activity, enhanced cellular uptake, and restricted tumor growth in tumor-bearing mice while minimizing drug-associated toxicity. Similarly, Lu et al., 2018 synthesized a polymeric amphiphilic ionophilic micelle having an ultralow CMC and superior serum stability. The formulation greatly enhanced doxorubicin (DOX) uptake at tumor sites, resulting in effective melanoma eradication and reversal of tumor growth in mice. In another report, Zhu et al., 2020 designed a theranostic micellar system (Ce6-CSPD/DOX) comprised of chitosan and hydrophobic polymethylacrylamide derivatives having Chlorin e6 (Ce6) and DOX. This multifunctional platform concurrently induced cancer cell apoptosis while restoring fluorescence and photoactive properties, supporting both therapy and imaging. Targeted micellar systems have also shown considerable ability. A PLGA-PEG-retinoic acid (RA) polymeric micelle was fabricated for the targeted administration of irinotecan to HT-29 human colorectal cancer and HepG2 cells. The targeted formulation displayed significantly greater cytotoxicity than both non-targeted micelles and free irinotecan (Emami et al., 2018). Similarly, PEG-poly (beta-amino ester) (PBAE)-PEG triblock copolymer micelles were developed for pH-reactive delivery of the hydrophobic anticancer drug verteporfin. The morphology of micelle can be tuned by adjusting the hydrophobicity of the PBAE core segment that reduce the macrophage uptake while maintaining potent anticancer property against human triple-negative breast cancer (TNBC) and small-cell lung cancer tissue (Kim et al., 2018). Combination of PEG with micelle may hinder cellular uptake which can be eliminated by developing cRGD peptide ligand-decorated PEG–poly (L-lysine) (PEG–PLL) micelles for delivering antiangiogenic plasmid DNA. The DNA was attached to the micellar core through electrostatic interactions and the resulting nanocarrier showedimproved tumor-cell uptake, remarkable transfection efficiency and substantial inhibition of BxPC3 tumor development *in vivo* (Dirisala et al., 2014). Along with drug delivery, PMs have also been used for improving tumor penetration and imaging applications. Inoue et al., 2021 reported that uniting TGF-β and chloroquine with nano-eruptions in GFP-labeled pancreatic tumors resulted in higher micellar diffusion through the tumor stroma. This effect was achieved through increased diameter of tumor vessel brought by chloroquine and enhanced dynamic valve creation mediated by TGF-β, resulting in enhanced drug delivery efficiency. Additionally, PEG–PLL conjugated PMs have demonstrated substantial tumor accumulation and enhanced T1 imaging performance, highlighting their potential as theranostic agents (Shiraishi et al., 2010).

#### 3.2.4 Dendrimer

Dendrimers are basically polymeric macromolecules with branched, three-dimensional structure gaining importantinterest in cancer nanomedicine owing to their unique architecture, monodispersity and adjustable physicochemical properties. Structurally, a dendrimer composed of three basic components: a central core, repetitive branching unitsand several terminal functional groups on the surface. Their stepwise synthesis permits precise size control, molecular weight, branching configuration and surface functionality. These distinguish them from conventional polymers formed via random polymerization. The dendrimers are spherically shaped, size ranging from 1.5 to 14.5 nm, high multivalency and a huge number of surface groups that isused for specific biomedical applications. These unique structural properties make them suitable for drug delivery platforms (Zenze et al., 2023). Therapeutic agents are bound to dendrimers within the internal cavities via hydrogen bonding, hydrophobic interactions, or chemical bonding, or they can be covalently bonded to the core or terminal surface groups. The highly branched structure provides feasible attachment of targeting ligands, imaging agents and therapeutic molecules. Moreover, dendrimers can be synthesized with biodegradable backbones and controlled surface characteristics, permittingoptimized drug solubility, stability, release kinetics, pharmacokinetics, and biocompatibility (Newkome et al., 2001). The chemistry involved in dendrimer preparationsignnificantlyinfluences their performance, including solubility, degradability and therapeutic efficacy. Some of the dendrimers used in the biomedical applications contains polyamidoamine (PAMAM), poly (propylene imine) (PPI), poly-L-lysine (PLL),carbohydrate-based dendrimersand polyamide-based dendrimers. Among these, PAMAM are the most widely explored and are commercially available in multiple generations with diverse surface functionalities (Wróbel et al., 2023). A major advantage associated with dendrimers is their capaity to improve the solubility and bioavailability of weakly water-soluble anticancer drugs. For example, Rai et al., 2024 combined chrysin within fucose-decorated PAMAM dendrimers and examined its anticancer property against human lung cancers. When combined with dendrimer, it markedly increased chrysin solubility, exhibiting faster dissolution rates and supeior drug release in both acidic and neutral media than the free drug. Dendrimers can also facilitate oral drug delivery across intestinal barriers. Similarly, N-acetyl-D-glucosamine (NAG)-combined PAMAM dendrimers were used to improve the transfer of camptothecin to A549 lung cancer cells stating lectin receptors and glucose transporters. The NAG-modified dendrimer showed∼ 4.5-times greater cytotoxicity than bare camptothecin, underlining the use of receptor-mediated targeting (Pooja et al., 2020). However, the PAMAM dendrimers lacking behind the *in vivo* application due to the positively charged amine groups present on PAMAM, which may induce cellular toxicity under physiological conditions. To address this drawbacks, surface functionalization strategy like PEGylation has been used to increase biocompatibility, lowering toxicity, extending blood circulation time and improving systemic stability (Luong et al., 2016). Cancer treatment involves dendrimer–drug conjugates strategy where drug molecules are covalently bound to the dendrimer core, branching points, or usually, the terminal surface groups. The wide variety of peripheral functional groups leadslinking of multiple drug molecules, substantially increasing local drug accumulation at the target area. Controlled drug release can be resultedvia stimuli-responsive linkers, such as acid-labile or disulfide bonds, thatunderstands the acidic and reducing conditions characteristic of the tumor microenvironment. There are some literature that have successfully incorporated drugs into dendrimer systems. Lai et al., 2007 reported that dendrimer-based delivery of doxorubicin combined with photochemical internalization (PCI) technology helps in easy intracellular drug release and enhanced cytotoxicity in cancer cells. Cisplatin–dendrimer conjugates exhibited superior antitumor activity (*in vitro* and *in vivo*) compared with bare cisplatin in B16F10 tumor-bearing mice. Similarly, Zhuo et al., 1999 synthesized 5-fluorouracil (5-FU)-dendrimer conjugates using a time-sequenced propagation method that allowed favorable controlled-release property. PEGylated dendrimer–doxorubicin conjugates expanded the blood circulation, limited systemic toxicity, decreased drug accumulation in healthy tissues and restricted the development of doxorubicin-unresponsive C-26 tumors in mice. Also, PAMAM functionalized with PEG conjugated with 5-FU showed sustained drug release *in vitro* and *in vivo*, while also lowering drug leakage and hemolytic toxicity (Bhadra et al., 2003).

Besides being a potential nanocarriers, dendrimers have shown significant possibility in gene delivery, vaccine development, immunotherapy, magnetic resonance imaging, tissue engineering, and antiviral, anticancer, antibacterial applications due to their distinct structure, controllable synthesis, high loading capacity, and versatile surface functionalization.

### 3.3 Some other reported advanced smart nanocarriers in cancer therapy

In addition to the already discussed nanocarriers, there are some recently developed nanoplatforms that are gaining interest in the field of cancer immunotherapy due to their unique physicochemical properties and extraordinary therapeutic potential. These includes black phosphorus (BP), metal–organic frameworks (MOFs).

#### 3.3.1 Black phosphorus

Black phosphorus (BP) was initially developed in 1914 and thereafter it has attracted considerable scientific attention in biomedical research due to its distinctive physical, chemical and biological properties (Bridgman, 1914). The structure of BP comprise phosphorus atoms in an sp^3^ hybridized state, creating puckered layers stacked through weak van der Waals bond which make it the most steady allotrope of phosphorus. Different methods are employed for their production that includes mineralization, high-pressure conversion and mechanical milling methods. Among these, high-energy mechanical milling (HEMM) is the commonly used approach for synthesizing BP as it form biocompatible and biodegradable nanomaterials in a simple way that are suitable for biomedical applications (Wu et al., 2019). The high suitability of BP in cancer photoacoustic (PA) imaging and photothermal therapy (PTT) lies in their excellent photothermal conversion efficiency under near-infrared (NIR) irradiation (Deng et al., 2018). For instance, a solvent-free HEMM method has been used to synthesize water-soluble, PEGylated BP nanoparticles with high yield. These nanoparticles displayed uniform size distribution, enhanced biocompatibility, outstanding photostability and efficient NIR-induced heat production. It makes it highly efficient as multifunctional nanotheranostic agents for collective cancer imaging and photothermal treatment (Sun et al., 2016).

#### 3.3.2 MOF as nanocarriers

Metal-organic frameworks (MOFs) are the class of hybrid porous substances that are composed of metal ions or clusters coordinated with organic linkers. A variety of MOFs has been synthesized recently based on their highly tunable chemical composition including zeolitic imidazolate frameworks (ZIFs), alkaline earth metal-based frameworks (AEPFs), Materials Institute Lavoisier (MIL) structures and rare-earth-based frameworks (RPFs). Some of the commonly used synthesis methods for MOFs are mechanochemical, solvothermal, coprecipitation, microwave-assisted and sonochemical (Oliva González et al., 2021).

Owing to the distinctive structural properties of MOFs like their adaptable pore size and geometry, exceptionally extended surface area and versatile functionalization capabilities, they are attractive candidates for drug delivery. Their biomedical application has been restricted by issues related to physiological instability and potential cytotoxicity associated with certain metal ions. MOFs engineered to integrate with other functional materials serve as improved multifunctional nanoplatforms for cancer treatment. These includes photodynamic therapy (PDT), PTT, chemotherapy, immunotherapy and combination therapies (Wuttke et al., 2017). As an example, in a recently reported study, an endogenous copper-based MOF nanoenzyme was found to enhance the synergistic effects of hydrogen sulfide (H_2_S)-activated near-infrared (NIR) photothermal therapy and chemodynamic therapy for colon cancer treatment. This biomarker-responsive “turn-on” strategy produces therapeutics directly without requiring additional drug cargo *in situ*, thus making the design of nanoparticle simpler while offering a promising approach for targeted colon cancer therapy (Li et al., 2020).

Beyond the compositional classification of nanocarriers, their biological performance is strongly governed by their physicochemical characteristics. Hence, characterization of micelles, vesicles/liposomes, polymeric nanoparticles, inorganic nanoparticles, and hybrid nanocarriers are carried out thoroughly to identify the particle size, size distribution, morphology, surface charge, surface chemistry, drug-loading/encapsulation efficiency, release behavior and other (He et al., 2010). The morphology and particle size is commonly studied using techniques like transmission electron microscopy (TEM), scanning electron microscopy (SEM), atomic force microscopy (AFM) and dynamic light scattering (DLS). Determining zeta potential gives information about surface electrostatic properties and other spectroscopic and chromatographic techniques like FTIR, XRD, XPS, NMR, DSC helps to identify the surface functionalization, drug-carrier interactions, drug loading and release (Herd et al., 2013). As an example, characterization of polymeric nanocarriers demonstrated that particle size and surface charge can significantly modify the cellular uptake and biodistribution of drugs, though the extent of these effects depends on composition of nanoparticle and biological system. Similarly, nanoparticles with comparable composition but having different geometry and shapes can highly influence cellular internalization and uptake mechanism (Herd et al., 2013). Experimental studies on surface charge of nanocarriers highlights that change in its surface functionalization can differ formed protein corona composition and subsequent cellular association, while specific corona proteins may either promote or suppress cellular uptake (Ritz et al., 2015). Hence, optimization of nanocarriers based on the physochemical parameters such as size, morphology, surface charge, surface property can significantly improve the biodistribution, tissue penetration, cellular internalization, intracellular trafficking, and therapeutic exposure. Specifically in cancer nanomedicine, studies on these parameters are highly essential as properties that favor cellular uptake not always maximize penetration throughout solid tumors. For example, recent study on 3D tumor model revealed that NPs with larger size performed greater cellular internalization but poorer penetration into tumor spheroids, underlining the trade-off between cellular uptake and tissue penetration (Cybulski et al., 2025).

Apart from the primary role of cellular targeting and enhanced bioavailability of therapeutic agents, nanocarriers are also systematically engineered to accumulate drugs having varying physiochemical properties to release at the targeted site. This feature is particularly important for hydrophobic drugs, which generally experience poor aqueous solubility and consequently face challenges for conventional drug delivery. Hydrophobic drugs can be incorporated into nanocomposites through several interactions like non-covalent adsorption, hydrophobic interactions, π–π stacking, hydrogen bonding, coordination interactions, or physical encapsulation within hydrophobic domains and porous structures. Depending on the type of nanocarrier, hydrophobic drugs can be loaded on graphene-based surfaces, encapsulated within polymeric/lipid substances, or accumulated in the pores of mesoporous inorganic components. In case of graphene-derived nanocarriers, the presence of oxygen-containing functional groups can further facilitated hydrophobic drug loading; for example, GO, rGO, GQDs were reported to exhibit high curcumin loading, with pH-dependent release and enhanced anticancer activity (Some et al., 2014). A recent study about GO based nanocarrier demonstrated that a ZnO-GO nanocomposite can act as a carrier for the hydrophobic anticancer drug paclitaxel with enhanced drug delivery and cytotoxicity toward breast cancer cells (Madeo et al., 2024). Similarly, Sultana et al., 2025 reported a nano-MOF–GO composite that encapsulate the hydrophobic drug piperine. In this study, incorporation of GO increased drug-loading sites and reduced premature drug leakage under physiological conditions. In another study, PLGA-coated magnetic nanoparticles (PLGA-MNPs) was developed that enabled the co-encapsulation of hydrophobic paclitaxel/rapamycin with hydrophilic carboplatin (Schleich et al., 2013). The HER2-targeted formulations further boosted cellular cytotoxicity and apoptosis, highlighting the potential of combining controlled release, active targeting, and multidrug delivery. Drug release can be further controlled using pH-, redox-, enzyme-, light-, or ROS-responsive mechanisms so that the drugs release at targeted site. In relation with metal-oxide-containing nanocomposites, Eixenberger et al., 2025 demonstrated that paclitaxel loaded-ZnO nanoparticles in lipid were able to generate ROS under UV irradiation, which then resulted in lipid peroxidation, disruption of the carrier, thereby triggering drug release. This showed the triggered drug delivery system exhibited more cytotoxic activity than the non-triggered one on T47D breast cancer cells.

The major characteristics of drug-loaded nanocarriers in terms of drug delivery, release, drug half-life, stability, cytotoxicity, clinical translation, model, evidence stage, regulatory statusare summarized in Table 1.

**TABLE 1:** Major characteristics of drug loaded nanocarriers.

Type of nanocarrier	Example of drug	Drug delivery/ payload suitability	Mechanism of drug release	Drug half-life/ circulation	Stability	Cytotoxicity/ safety	Clinical translation	Model/ Indication	Evidence stage	Regulatory status
Gold nanoparticles (AuNPs)	Doxorubicin, paclitaxel	Small molecules, nucleic acids; targeted and theranostic delivery	pH-, redox-, enzyme-, NIR- and photothermal-triggered release	Depends on size, coating and surface chemistry; may prolong circulation	Good with suitable surface coatings; aggregation may occur	Dose-, size- and surface-dependent; concerns regarding long-term accumulation	Mainly preclinical; limited clinical translation of AuNP-based drug-delivery systems	*In vitro* and *in vivo* cancer models; experimental solid tumors	Preclinical	Investigational; no approved systemic anticancer AuNP drug-delivery product
Silver nanoparticles (AgNPs)	Doxorubicin, 5-FU	Drug delivery combined with intrinsic antimicrobial and anticancer activity	Diffusion, pH-responsive release, bond cleavage and Ag^+^ release	Strongly dependent on particle size and coating; often subject to MPS uptake	Oxidation and aggregation can affect stability	Greater toxicity concern due to Ag^+^ release and ROS generation	Predominantly preclinical; limited clinical translation for systemic anticancer delivery	*In vitro* and *in vivo* cancer models	Preclinical	Investigational; no established approved systemic anticancer AgNP drug-delivery product
Platinum nanoparticles (PtNPs)	Doxorubicin, cisplatin	Drug delivery with catalytic/ROS-modulating and therapeutic functions	pH-, redox- and enzyme-responsive mechanisms	Dependent on size and surface functionalization	Relatively chemically stable; aggregation may occur	Dose-, size- and surface-dependent; long-term fate requires evaluation	Predominantly preclinical; clinical translation remains limited	*In vitro* and *in vivo* cancer models	Preclinical	Investigational
Iron oxide nanoparticles	Doxorubicin, paclitaxel	Magnetic targeting, drug delivery, MRI and magnetic hyperthermia	pH-, magnetic-, enzyme- and heat-responsive release	Can provide prolonged circulation with appropriate coatings	Good with suitable coatings; aggregation can occur	Generally favourable with appropriate formulations; iron metabolism must be considered	Relatively advanced; several iron-oxide nanoparticle formulations have reached clinical use, although drug-delivery applications remain less mature	*In vitro*, *in vivo* and clinical applications; imaging, iron replacement and experimental drug delivery	Preclinical to clinical; formulation-dependent	Selected iron-oxide products approved; anticancer drug-delivery formulations largely investigational
Carbon nanotubes (CNTs)	Doxorubicin, paclitaxel	High-capacity delivery of hydrophobic drugs; photothermal and targeted delivery	Diffusion, π–π interaction disruption, pH-, NIR- and redox-responsive release	Potentially prolonged retention; clearance can be limited	High structural stability; dispersion is challenging	Concerns include oxidative stress, inflammation and biopersistence	Mostly preclinical; significant toxicity and regulatory concerns limit translation	*In vitro* and *in vivo* cancer models	Preclinical	Investigational
Graphene oxide (GO)	Doxorubicin, paclitaxel	High loading of aromatic/hydrophobic drugs; photothermal and targeted delivery	π–π interaction disruption, pH-, redox-, NIR- and enzyme-responsive release	Dependent on size and functionalization; possible prolonged retention	Good structural stability; aggregation may occur	Dose-, size- and surface-dependent; potential oxidative stress and accumulation	Predominantly preclinical; clinical translation remains limited	*In vitro* and *in vivo* cancer models	Preclinical	Investigational
Liposomes	Doxorubicin, daunorubicin, paclitaxel	Hydrophilic and hydrophobic drugs; highly versatile	Diffusion, membrane destabilization, pH-, enzyme- and temperature-responsive release	Can substantially increase circulation compared with free drug	Generally good; leakage and oxidation may occur	Generally favourable; depends on lipid composition and formulation	Highly clinically advanced; several liposomal drug products are approved for clinical use	*In vitro*, *in vivo* and human clinical studies; cancer indications include ovarian cancer, AIDS-related Kaposi sarcoma, multiple myeloma and AML depending on formulation	Preclinical to phase III and post-marketing; formulation-specific	Several formulations approved; post-marketing clinical use established
Solid lipid nanoparticles (SLNs)	Paclitaxel, doxorubicin	Particularly suitable for poorly water-soluble/lipophilic drugs	Diffusion and lipid-matrix degradation; sustained release	Can prolong circulation and reduce rapid elimination	Generally good; drug expulsion during storage can occur	Generally favourable because of biocompatible lipid components	Early-to-moderate clinical translation; mainly investigated in preclinical and clinical-development studies	*In vitro* and *in vivo* cancer models; selected clinical-development studies	Preclinical to early clinical	Investigational
Polymeric micelles	Paclitaxel, docetaxel	Particularly effective for poorly water-soluble drugs	Diffusion, micelle disassembly, hydrolysis, pH- and redox-responsive release	Can improve circulation and reduce premature drug elimination	Stability can be limited after dilution below CMC	Generally favourable depending on polymer composition	Relatively advanced; several polymeric-micelle formulations have entered clinical development, with some reaching clinical use	*In vitro*, *in vivo* and clinical cancer studies; formulation-specific indications	Preclinical to phase I–III; formulation-specific	Mostly investigational; selected formulations have reached clinical use/approval
Dendrimers	Methotrexate, doxorubicin, siRNA	Drugs and nucleic acids; precise surface functionalization and targeting	Hydrolysis, enzymatic cleavage, pH- and redox-responsive release	Can be prolonged depending on generation and surface modification	High structural and formulation stability	Cationic dendrimers may cause membrane damage; surface modification can reduce toxicity	Mostly preclinical/early clinical development; limited clinical adoption	*In vitro* and *in vivo* cancer models; selected early clinical candidates	Preclinical to early clinical	Investigational
Black phosphorus	Doxorubicin, paclitaxel	Drug delivery combined with photothermal/photodynamic therapy	pH-, NIR- and degradation-responsive release	Dependent on surface modification and degradation	Poor intrinsic aqueous/oxidative stability; protective coatings often required	Generally promising degradation profile, but dose and oxidative effects require evaluation	Predominantly preclinical; clinical translation is at an early stage	*In vitro* and *in vivo* cancer models	Preclinical	Investigational
Metal–organic frameworks (MOFs)	Doxorubicin, 5-FU, ibuprofen	High-capacity delivery of small molecules, proteins and combination payloads	Pore diffusion, ligand exchange, pH-, enzyme-, redox- and ion-responsive degradation	Strongly dependent on size, composition and surface modification	Highly tunable but stability varies with metal–ligand chemistry	Depends on metal/ligand composition and degradation products	Emerging platform; predominantly preclinical with substantial translational challenges	*In vitro* and *in vivo* cancer models	Preclinical	Investigational

### 3.4 Nanoengineered cancer vaccines

The idea of nanovaccine comes out as major step up from the conventional vaccine as it takes the advantage the utilization of nanotechnology to enhance and refine immunization strategies. These vaccines use nanocarriers (NC) deliver antigens, adjuvants and other immunomodulatory agents with improved accuracy and efficiency. The physicochemical charactersitics of these nanocarriers, including their shape, size, surface properties and chemical composition can be designed to optimize antigen delivery, increase immunogenicity and achieve targeted immune activation. Usually, there are three important components that make up a typical nanovaccine, namely antigens, adjuvants, and nanocarriers that together work to initiate, amplify and sustain immune responses against disease-specific targets, especially cancer cells (Gurunathan et al., 2024). Based on their design and mechanism of action, nanovaccines can be broadly divided into four types-neoantigen nanovaccines, STING agonist-based nanovaccines, artificial antigen-presenting cell (APC) nanovaccines and RNA-based nanovaccines (Figure 4).

**FIGURE 4:**
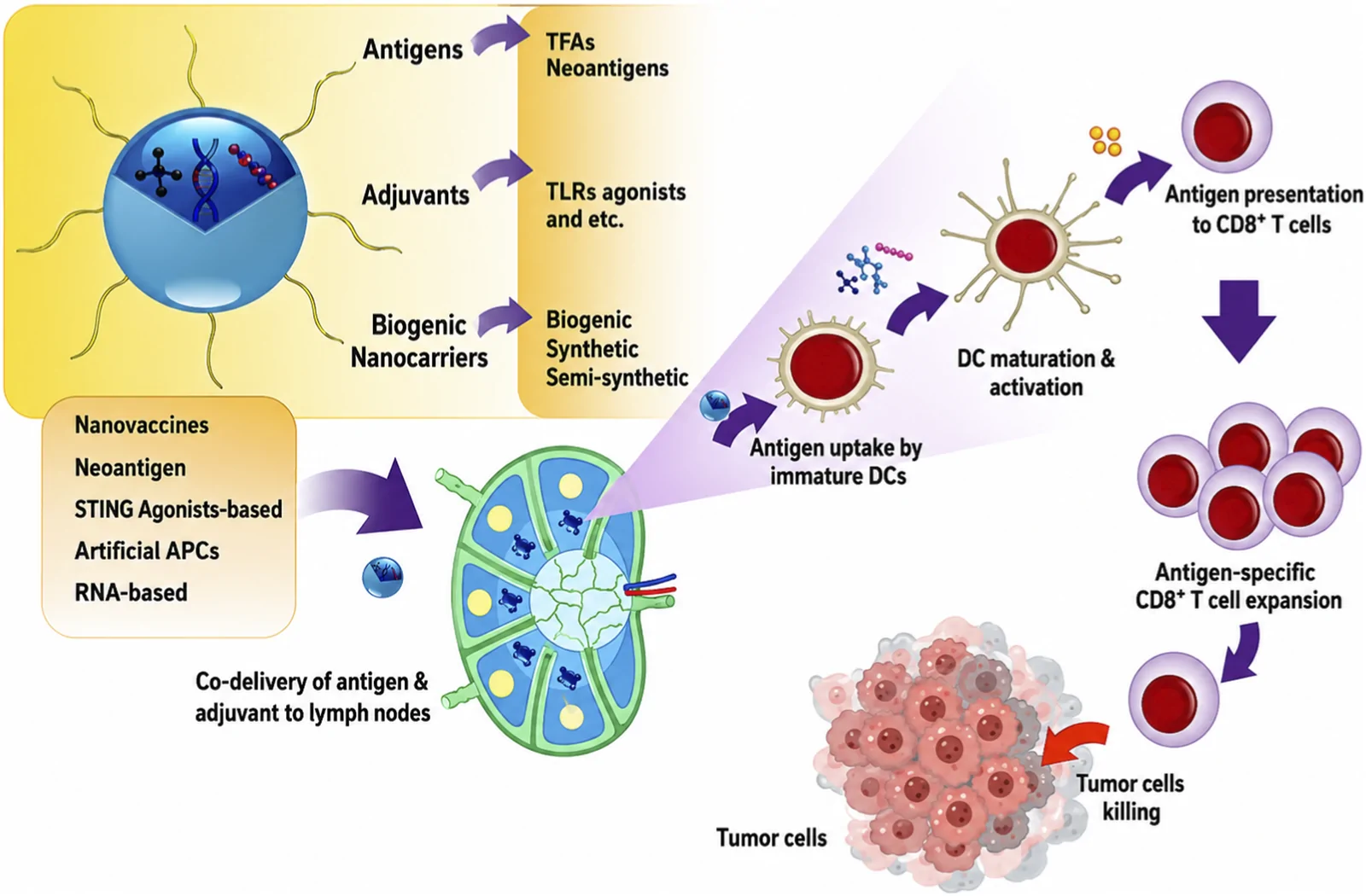
Representation of general structure of nanovaccines, types and mechanism of action in cancer therapy (adapted from Fang et al., 2022).

### 3.5 Neoantigens and neoantigen-based vaccines

Neoantigens, are tumor-specific antigens developed due to genetic alterations, especially somatic mutations and are not encountered in healthy cells. Besides mutated proteins, dysregulated RNA transcripts and virally encoded proteins can also act as sources of neoantigens. The use of neoantigens in cancer immunotherapy has been considered as promising option due to their selective expression in tumors and can be recognized by T lymphocytes upon exhibition via major histocompatibility complex (MHC) molecules. The generation of neoantigens may also come from viral open reading frames in virus-associated malignancies, such as EBV-derived nasopharyngeal carcinoma and HPV-positive cervical cancer (Wang and Cao, 2020).

Neoantigens can be categorized into two types-shared neoantigens and personalized neoantigens, based on their occurrence. Shared neoantigens result from recurrent mutations found in numerous patients and may act as candidates for broad-spectrum therapeutic cancer vaccines. Personalized neoantigens, on the other hand, are specific for each particular tumor type and serve as targets for tailored immunotherapy treatment (Zhou et al., 2020). Detection of Neoantigens is based on the sequencing of genomic of both cancerous and healthy tissues, followed by bioinformatic examination to identify somatic mutations and predict MHC-I/II-binding neoepitopes. Proteomic analysis based onmass spectrometry (MS) can be used for the verification of identified neoantigens. After examination of immunogenicity and synthesis of selected peptides, these neoantigens are integrated to therapeutic vaccines. There are number of neoantigen-based cancer vaccines made using DNA, RNA, and peptides (Blass and Ott, 2021). Though highly specific, one critical drawback of neoantigens is their poor immunogenicity. The development of nanovaccines can be considered as one of the most efficient ways to optimize the delivery of neoantigens, their precise targeting and activation of immune response. One example is the sHDL-based nanodiscs for delivery of peptides via disulfide linkage. Combination of these nanodisc with cholesterol modified adjuvants make them more efficient at draining lymph node targeting and elicitation of antigen-specific CD8^+^ T-cell response (Kuai et al., 2017). Based on experiments conducted in melanoma bearing mice, preclinical trials revealed that there was significant increase in antitumor immunity due to the administration of neoantigen nanovaccines. A ten-times enhancement in the number of neoantigen-specific T cells than free vaccines as well as an elevation in the production of TNF-α and IFN-γ were observed. In addition, average time of tumor reoccurrence was found to increase from 11 to 16 days, with an extended median existence of around 8 days. Improved cytotoxic activity of splenic lymphocytes was also detected, representing stronger systemic antitumor responses (Chu et al., 2022). These findings indicate antitumor immune memory-related protection against recurrence; however, evidence from such studies remains predominantly preclinical, and assessment of metastatic control is not consistently included.

Despite encouraging preclinical results, the clinical translation of neoantigen-based nanovaccines remains challenging because of substantial antigen heterogeneity, tumor evolution and immune escape. Personalized neoantigens are patient-dependent and their detection depends on tumor sequencing, bioinformatic prediction, and experimental validation, resulting in considerable manufacturing time and complexity. Moreover, neoepitope presentation is HLA-dependent, which means an antigen predicted to be immunogenic in one patient may not be effectively resulted in another, thereby limiting the broad applicability of personalized vaccines. Tumor heterogeneity further permit antigen-negative clones to escape immune recognition, while ongoing tumor evolution can alter the neoantigen selection during treatment. Consequently, personalized vaccine design, synthesis, quality control and delivery need patient-specific manufacturing and considerable time and resources, which can be difficult to reconcile with rapidly progressing cancers. These drawbacks highlight the need for rapid neoantigen identification, standardized manufacturing platforms, improved prediction of clinically relevant epitopes, and nanocarriers capable of co-delivering antigens and appropriate adjuvants. Thus, thoughthe most current nanovaccine evidence remains preclinical, overcoming these biological and manufacturing barriers will be essential for translating personalized neoantigen nanovaccines into clinically scalable therapies. To avoid this, the blending of nano vaccines with immune checkpoint inhibitors has been considered quite helpful (Türeci et al., 2018).

### 3.6 STING agonist–based nanovaccines

STING stands for stimulator of interferon genes, a motioning protein allied with endoplasmic reticulum and is important for innate and adaptive immunity. Once cytosolic DNA molecules are identified by the enzyme cyclic GMP-AMP synthase (cGAS), activation of STING triggers in the manufacturing of type 1 IFNs as well as pro-inflammatory cytokines. This promotes pathogen killing and antitumor immunity (Amouzegar, et al., 2021). Apart from innate immunity, STING signaling is also related to adaptive immunity in DNA vaccines. In respect of cancer immunotherapy, the role of STING signaling is to enhance antigen presentation and priming of T-cells. STING signaling allows CD8^+^ DCs to secrete type I IFNs, thus allowing cross presentation of antigens and activation of CD8^+^ T cells. Moreover, B cells and CD11b^+^ tumor-infiltrating antigen-presenting cells (APCs) can identify tumor-mediated STING-activating elements that helps to the production of leukocyte-derived type I IFN and initiation of natural killer (NK) cells for tumor cell elimination. STING signaling also initiate the spontaneous and radiotherapy-induced antitumor T-cell responses (Ng K. W. et al., 2018; Ng S. L. et al., 2018).

The involvement of nanotechnology for therapeutic improvement of STING agonists, particularly cyclic dinucleotides (CDNs), enhance their cytosolic delivery and bioavailability. In these formulations, the STING agonist basically plays as an immunostimulatory adjuvant rather than as a tumor antigen, and the therapeutic effect depends on activation of innate immunity and subsequent enhancement of tumor-antigen presentation. As an example, cyclic di-GMP encapsulated PEGylated lipid nanoparticle prompted enhanced activation of CD4^+^ and CD8^+^ T cells than bare CDNs (Hanson et al., 2015). This represents a preclinical nanovaccine aspproach, with efficacy demonstrated in experimental models rather than clinical studies. Additionally, an enhanced infiltration of CD8^+^ T cells was observed when the STING activator PC7A nanovaccine was delivered intratumorally to melanoma bearing mice. This resulted from increased production of CXCL9 by the myeloid cells due to accumulation of PC7A nanovaccine in the tumors (Jiang X. et al., 2022). This in turn, stimulate recruitment of IFN-γ–producing CD8^+^ T cells from peripheral tissues into the TME. The example of increasing STING activation using additional nanoparticle formulations can be seen when CDNs have been incorporated into pH-responsive endosomolytic polymersomes and STING-activated pH-responsive polymer nanovaccines. This support the cytosolic delivery by simplifying endosomal escape and exposing nucleic acids within the cytoplasm. These strategies intensify STING signaling and strengthen antitumor immune responses (Hanson et al., 2015).

A major downstream effect related to STING activation is the induction of type I IFN signaling, which is mediated through the interferon-α receptor (IFNAR) and initiates pathways such as JAK–STAT. The tumor angiogenesis can be resulted due to the increased cytotoxicity of NK cells by Type I IFNs, promote differentiation and maturation of APCs, support priming of T-cell, increase MHC class I expression on tumor cells and restrict vascular endothelial growth factor (VEGF) production (Yang et al., 2018).

Activation of STING also impacts the tumor vasculature. Signaling through STING enhances vascular perfusion and promotes the production of adhesion molecules like E-selectin, VCAM-1, and ICAM-1 that help the movement of tumor-infiltrating lymphocytes. Higher expression levels of STING in endothelial cells is responsible forincreased infiltration of T-cells and in improvement of patient survival in colon and breast cancers (Yang et al., 2019). Nevertheless, the therapeutic effect is found to be dose-dependent, in the sense that while lower amountsof STING agonists such as cGAMP and ADU-S100 normalize vasculature and augment CD8^+^ T-cell infiltration, higher doses such as DMXAA may lead to vascular necrosis and tumor bleeding, even impair immune-cell recruitment and function within the TME (Matthews et al., 2006).

### 3.7 Artificial APCs

Antigen-presenting cells (APCs) act as a connection between innate and adaptive immunity and it is mostly a preclinical/early translational approaches. Once the antigens are taken up by APCs, they are processed by APCs and show peptide fragments on major histocompatibility complex (MHC) with costimulatory signals and hence activate antigen-specific T cells and initiate adaptive immune responses. DCs, macrophages, and B cells are the primary professional APCs; however, other cell types like keratinocytes, fibroblasts, and endothelial cells may also present antigens in certain condition (Banchereau and Palucka, 2005). Among them, DCsare of significant importance in cancer immunotherapy owing to their capacity for T-cell priming. The autologous DC vaccines are formed by the separation of autologous DCs from patients, followed by antigen loading or stimulation and re-injection into patients to induce antitumor immunity. This strategy has proved to be clinically effective with the approval of PROVENGE (Sipuleucel-T) by the U.S. Food and Drug Administration in 2010 for prostate cancer recovery. However, the therapy is constrained by the requirement for personalized production, expensive, laborious procedure and scarce cellular source (Cheever and Higano, 2011). To overcome these challenges, engineered artificial antigen-presenting cells (aAPC), is being used as a synthetic platform capable of mimicking the critical interactions between DCs and T cells. aAPCs present cognate antigenic peptides with respect to MHC molecules for recognition by T-cell receptors (TCRs), while simultaneously showingcostimulatory ligands that bind receptors at the immunological synapse. aAPCs have an advantage over natural APCs as they are of controllable composition, even signal presentation, scalable manufacturing and the potential for ready-to-administer vaccine formulations (Sunshine and Green, 2013).

aAPCs can be broadly divided into two main approaches: genetically modified cellular aAPCs and biomaterial-based synthetic aAPCs. Genetically engineered cells such as K562 human leukemic cells and NIH/3T3 murine fibroblasts have been involved to activate cytotoxic CD8^+^ T cells by expressing MHC and costimulatory molecules. In contrast, bioengineering strategies have resulted synthetic aAPCs through the use of nanoparticles, microparticles, and other biomaterial scaffolds effective to deliver tuned activation signalsprecisely (Hasan et al., 2016).

The physicochemical properties of aAPCs-viz., size, shape, and surface functionalization—greatlyaffect T-cell activation, proliferation and therapeutic performance. Consequently, engineering these parameters are considered vital for optimizing immunomodulation. Biomaterial-based aAPCs are already acknowledged for their ability to induce antitumor immune responses both *in vitro* and *in vivo* (Turtle and Riddell, 2010). Recent studies demonstrated attempts to convert tumor cells themselves into aAPCs. One example is the using of herpes simplex virus 1-loaded oncolytic virus that encodes IL-12 and TNFSF4 (OX40L) to infect tumour cells and induce APC-like stimulatory functions. These engineered cells expressed APC-related markers, stimulates antigen-specific T cells and increased T-cell cytotoxicity in co-culture with tumor-infiltrating lymphocytes (TILs) (Ye et al., 2022). In mouse tumor models, combination of OV-OX40L/IL-12and TIL therapy produced complete tumor deterioration, generated durable antitumor immune memory, and reprogrammed tumor-associated macrophages toward an M1 phenotype (Ye et al., 2022).

### 3.8 RNA based nanovaccine

RNA nanovaccines are predominantly preclinical therapeutic studies in melanoma/colon cancer models and serve as one of the innovative ways to treat cancers through the administration of RNA molecules such as messenger small interfering RNA (siRNA), RNA (mRNA) and microRNA (miRNA) into tumor cells. Among these, mRNA vaccines have become more widespreadfor controlled protein expression without the peril of genomic integration associated with DNA-based therapies. Moreover, mRNAshow higher transfection efficiency, particularly in immune cells and can be developed efficiently *in vitro* (Lin et al., 2020). However, RNA molecules alone are naturally unstable, having a short half-life and being very susceptible to destruction by nucleases. In order to resolve this issue, nanocarriers such as lipid nanoparticles, polymeric nanoparticles, inorganic nanomaterials and bio-inspired nanovehicles are used to encapsulate and protect RNA molecules from nucleases and subsequent immune system attack. These nanocarriers also help in delivering RNA molecules APCs and immune cells within the tumor microenvironment, hence enhancing cellular uptake and therapeutic efficiency (Aagaard and Rossi, 2007; Tan and Sun, 2018). One example is the development of an RNA nanovaccine, “mRNA@lipoplex” using lipoplexes for the effective transport of mRNA to dendritic cells (DCs). The nanovaccine successfully facilitated mRNA transport into DCs, stimulated release of cytokines including IFN-γ, IL-12 and IL-2 stimulated antigen-specific CD8^+^ T cells and increased antitumor immune response (Hongxia et al., 2019). During early examination in preclinical melanoma and colon cancer models, the nanovaccine slowed down tumor growth, extended survival rate and established long-term immunological memory. Thus, RNA nanovaccines offer several benefits through their combination of RNA therapy and nanotechnology techniques (Fang et al., 2022).

Some recently developed nanovaccines used in cancer therapy, cancer type, their mechanism of action and results are shown in the following table (Table 2).

**TABLE 2:** Nanovaccines in cancer immunotherapy.

Nanovaccine	Type of cancer	Mechanism of action	Results	Ref.
BMT@LA (L-arginine)	bilateral U14- bearing tumor model in animal	➢ formation of singlet oxygen and nitric oxide ➢ enhancement of intracellular oxidative stress ➢ elevation of double-stranded DNA damage ➢ Promoting tumor cell apoptosis	Effects in tumor growth and metastatic progression, amplified CD8^+^ T-cell infiltration and facilitated the discharge of TNF-α, IL-6, and IL-12p70 hence enhancing antitumor immunity	Hongxia et al. (2019)
OMPN (OVA, MnO_2_, and polydopamine)	Orthotopic melanoma-bearing animal model (*in vitro* studies)	➢ elevating the movement and migration of DCs inthe inguinal lymph node ➢ Orienting anti-tumorimmune responses	Restricted tumor growth and liver metastasis, increased population of CD3^+^CD8^+^ T cells and helped M2 to M1 macrophage polarization. It also elevates IL-12p40 and IFN-γ points; lowering of IL-10 expression	Xiao et al. (2021)
F-PEI/OVA (OVA, fluoropolymer)	Orthotopic Melanoma and breast Cancer-bearing animal model (In vitro study)	➢ TLR4 used for DCs maturationvia signaling pathway ➢ Increasing antigentransportation into thecytosol of DCs ➢ Elevating antigencross-presentation	Inhibited post-surgical tumor redevelopment and metastasis, elongating survival up to 60 days	Xu et al. (2020)
banNV (Adpgk neoantigen, R848, CpG)	MC38 colorectal cancer Cell-bearing animal Model (*in vitro* study)	➢ Explaining PD-1 receptor on the surface of T cells ➢ vaccine uptake capacity enhanced by APCs	Improved the expression of the costimulatory molecules CD80, CD86 and CD40; stimulated APC-mediated secretion of IL-6, IL-12, and TNF-α, restricted tumor development thus extending the survival percentage to 70%	Ni et al. (2020)
(peptide, CpG oligodeoxynucleotides And cationic polymer Nanoparticle)	Breast cancer 4T1 Cells-bearing animal model (*in vitro* study)	➢ Creating more mature CD86^+^CD11c+ DCs ➢ Making strongvaccine-specific T cellimmune responses ➢ Increasing CD8^+^ T cellinfiltration tumor site, weakening thereappearance of local tumorand restricting metastasis tothe lungs	Combination therapy with PECT-Cur NPs and the nanovaccine improved systemic T-cell immune responses, limiting local tumor weight and recurrence and sufficiently inhibited lung metastasis	Liu et al. (2020b)
PLGA (calcinetin, R837)	Luc-4T1 cancer cell bearing animal model (*in vitro* study)	➢ Simultaneous delivery of calcinetin-expressed cancer cell membrane antigen andR837 adjuvant	Itformed a personalized antitumor immune response by increasingDC antigen uptake, reinforcing antitumor efficacy and initiating immune memory cells to give long-term protection	Xiong et al. (2021)
cMn-MOF@CM Mn-MOF, CpG, OVA	Melanoma B16 cancer cell bearing animal model (*in vitro* study)	➢ Increasing DC maturation and activation of T cell ➢ allevating tumor hypoxia ➢ Causing immunologic cell rupture ➢ Generating long-term immunogenic memory function	Itextended blood flow and increasedtargeting to tumor, enhanced the expression of CD80, CD86, and MHC II and activated the expansion of cytotoxic CD8^+^ T-cell subsets, thus forming TNF-α, granzyme B, IFN-γ and IL-2	Zhan et al. (2021)
Nanoprodrug (FIT NPs, tadalafil ICG photosensitizer)	CT-26 cells-colon cancer- bearing animal model (*in vitro* study)	➢ Directing MDSCs in the TME and strengthening tumor immunoreactivity ➢ Initiating maturation of DCs and T cells activation ➢ Amplifying tumorimmunoreactivity	It stimulated immunogenic cell rupture through the ICG photosensitizer and alleviated MDSC-mediated immunosuppression via tadalafil, henceuplifting photothermal immunotherapy. Strengthened antitumor immunity, enhanced PD-1^+^CD8^+^, CD8^+^granzyme B^+^and CD8^+^IFN-γ^+^ T-cell populations within tumors and suppresing tumor weight and size	Zhang et al. (2021)
PCO (PRT/CpG/OVA)	B16 melanoma Cells bearing animal model (*in vitro* study)	➢ The polycationic PRT results in improving PRT/CpG/OVAnanovaccine delivery ➢ DC’s antigen acceptance was increased and also the maturation	Blending of anti-PD-1 antibody and PRT/CpG/OVA enhanced immune checkpoint blockade capacity, hindered tumor immune escape, increased tumoricidal capacity, tumor-specific T-cell infiltration, and promoted BMDC activation through upregulation of CD80/CD86 hence, secretion of IL-6 and TNF-α	Jiang et al. (2022b)
OVAPEP-SLNP@CpG (lipid based, CpG, OVA)	Prophylactic and therapeutic, E.G7 tumor cell bearing animal model (*in vitro* study)	➢ Enlarging *in vitro* DC maturation, T cell cross-priming and antigen cross-presentation ➢ Elevating delivery, uptake of *in vivo* lymph nodeand DCs maturation	Results in exhibiting favorable therapeutic response following the initial nanovaccine immunization cycle and subsequent treatment with a second vaccination cycle cpupled with anti-Pd-1 therapy positively suppressed tumor relapse	Kim et al. (2020)
NTV (p [OEGMA4-DMAEMA22]-p [MA] 30 with conjugation ofNDP or PDP OVA241–27)	Human papillomavirus-E6/E7 And B16F10-OVA and tumor mice models (*in vitro* study)	➢ Changing the vaccinemorphology fromnanospheres intosheets in theendosomal environment ➢ Destroying the antigen’s delivery of endosomalmembraneto cytoplasm ➢ Leading anti-tumorimmune reactions ➢ Via re-assembly ofnanosheets by typical inflammation track activation	It induced a strong antitumor immune response with negligible systemic toxicity, while the combination of NTV and anti-Pd-L1 therapy extended animal survival and acquired tumor regression in nearly half of the treated mice	Gong et al. (2020)
LrTL (Trichosanthin, legumain, liposome)	Lewis’s lung cancer (LLC), B16-F10, intracranial LLC xenograft and CT-26 Colon cancer bearing animal model (*in vitro* study)	➢ Triggering DCs ➢ Discharging IFN-γ, TNF-α and IL-12 ➢ Empowering powerful CD8+T cell response ➢ Deleting of TAMs ➢ Restricting immunosuppressive effects remodeling of TME	It elicited a powerful antitumor immune response in both *in vitro* and *in vivo* models	Tang et al. (2021)
SeaMac (polymer NPs, neoantigen)	(CT26) and B16-F10 cancer cell bearing animal models (*in vitro*)	➢ Resulting maturation of DC ➢ DCs aggregation inlymph nodes ➢ Intensifying cytotoxic CD8+T cells	Itstarted a robust antitumor immune response, prompted abscopal effects in CT26 and B16-F10 tumor models and extended survival rate	Luo et al. (2021)
MSNs-ABC@PDAOVA (mesoporous silica NPs, OVA, polydopamine)	Melanoma bearing animal model (*in vitro* study)	➢ Identification of released antigen leading laserirradiation ➢ Increasing DCs maturationand activation ➢ Enabling migration of activated DCs to tumor-draining lymphnodes	Itformed strong antitumor immune responses and a single dose of MSNs-ABC@PDA-OVA combined with one round of photothermal therapy can sufficiently eradicated melanoma tumor cells. Also increased IFN-γ and TNF-α production, built robust immunological retentionand prevented tumor regeneration and lung metastasis	Huang et al. (2021)
PEI-modified GO transformable hydrogel (polyethylenimine, graphene oxide and R848-laden)	B16-OVA cells bearing animal model (*in vitro* study)	➢ Endorsed OVA mRNA expression while guarding it from degradation ➢ Initiated targeted delivery to lymph nodes ➢ Higher the population of antigen-specific CD8^+^ T cells ➢ Formed the production of antigen-specific antibodies	Suppressed tumor growth and metastatic spread	Yin et al. (2021)
hEX@BP (Black phosphorus quantum dots and exosomes)	LLC cells bearing animal model (*in vitro* study)	Promoted differentiationand maturation of DCs Increased infiltrating effector T cells into the tumor environment	The mixture of hEX@BP and photothermal therapy showed sustained photothermal efficacy, leading to rise in tumor temperature *in vivo*	Liu et al. (2020a)
LNT-NV (Lymph Node-targeted Neoantigen Nanovaccine having adjuvant Montanide)	B16F10 melanoma cells bearing animal model (*in vitro* study)	➢ Targets lymph nodes via amphiphilic liposomal delivery ➢ Increases uptake by CD11c^+^DC and endures DC maturation ➢ Promoteshigher neoantigen-definite CD8^+^ T-cell responses ➢ Elevates Th1 cytokine secretion (IFN-γ and TNF-α) and increased antitumor immunity	It exhibited ∼10-fold higher neoantigen-specific T-cell responses than bare vaccines, and its antitumor activity was further enhanced when coupled with anti-Pd-1 antibody or the Treg-inhibiting peptide P60. Successfully lowers tumor reoccurrence and delayed survival	Chu et al. (2022)
AECM@PC7A (antigen-Enriched cell membrane embedded PC7A nanovaccine)	B16-OVA melanoma cells bearing animal model (*in vitro* and *in vivo* study)	➢ IFN-γ develops tumor antigens on cancer cell membranes (AECM) ➢ PC7A triggers the STING pathway in antigen-presenting cells ➢ IncraesesDC’s antigen presentation and cross-dressing ➢ Boosted CD8^+^ T-cell-mediated antitumor immunity	This nanovaccineresulted significant tumor regression, restricted metastasis and formed long-term immune memory in multiple murine	Li et al. (2025)
OVA/Tp@Mn-DNA. (STING-activating cancer nanovaccine)	xenograft mouse cancer model (*in vivo* study)	➢ Offers antigen peptides to APCs via Mn^2+^–DNA self-assembled NPs ➢ Stimulates the cGAS–STING pathway, enabling IFN-β production ➢ SupportsDC’s maturation and antigen presentation ➢ Stimulates activation of CD8^+^ T-cell and antitumor immune responses	It effectively inhibited tumor growth and prolongedviability in tumor-containing mice showing great therapeutic potential for cancer immunotherapy	Tuo et al. (2026)

## 4 Scale-up, reproducibility, storage, and regulatory considerations

The clinical translation of nanomedicines needs precise control over critical quality attributes (CQAs), including particle size and size distribution, polydispersity index (PDI), morphology, surface charge, ligand density, drug loading, encapsulation efficiency, free-drug content, release kinetics, residual solvents, endotoxin, sterility, pH, and stability. These parameters can strongly influence colloidal stability, drug release, biodistribution, cellular uptake, and therapeutic performance. Hence, proper analytical approach, previously decided quality specifications, and quality-by-design approaches are essential for achieving reproducible nanomedicine products and establishing relationships between formulation/process parameters and biological performance. It is difficult to maintain these CQAs during scale-up as changes in mixing efficiency, heat and mass transfer, solvent evaporation, filtration, sterilisation, and particle-formation kinetics can result in variations in particle size, PDI, morphology, drug loading, and release kinetics. Differences in raw materials, equipment configuration, and process conditions can further contribute to batch-to-batch variability (Taha et al., 2020; Đorđević et al., 2022).

Storage stability highlights another important factor because nanocarriers can undergo aggregation, drug leakage, lipid degradation/oxidation and loss of structural integrity during storage. Freeze–thaw cycles can stimulate aggregation and loss of efficacy, while lyophilisation can cause particle aggregation, fusion, or payload leakage unless the formulation and drying process are properly optimized.

Multifunctionality possess another challenges for translation and regulation. While there are many advantages associated with nanoplatforms in preclinical studies that simultaneously deliver therapeutics, provide imaging, target specific cells, or modulate immune responses, however, more complex structure leads to the number of parameters requiring characterization and control. Additional functional components can introduce greater manufacturing variability, stability concerns, analytical requirements, and regulatory complexity. Therefore, multifunctionality is something that can increase therapeutic efficacy, but should be approached with proper caution (Hua et al., 2018; Taha et al., 2020).

## 5 Future perspectives

The current review underlines the prospects of nanomedicine for improving cancer therapy by overcoming the existing limitations of conventional treatments including nonspecific drug distribution, systemic toxicity, insufficient tumor exposure and less coordination of therapeutic and immune responses. Rationally designed nanocarriers can elevate the pharmacokinetics and target-specific delivery of drugs, while controlled or stimuli-responsive release and co-delivery strategies will provide possibility of coordinated chemo-, immune-, gene and phototherapy. Clinical test of liposomal doxorubicin has already proved that nanoformulation can retain antitumor efficacy while reducing treatment-related cardiotoxicity, demonstrating the potential of nanomedicine to improve the therapeutic index of established anticancer agents (Batist et al., 2001). In addition, preclinical nanovaccine reports further shows that nanoplatforms are capable of enhancing antigen delivery and activate tumor-specific T-cell responses, thereby providing opportunities to rectify immune suppression and improve therapeutic efficacy (Luo et al., 2017). However, these benefits cannot be generalized to nanomedicines as a whole, as heterogeneity in tumor and differences in nanoparticles accumulation in patient-to-patient can substantially influence therapeutic outcomes. Next-generation nanomedicine in cancer immunology has evolved as powerful tool to improve cancer diagnostic and treatment via targeted drug delivery, controlled release with minimum systemic toxicity. Numerous nanocarriers, including liposomes, polymers, dendrimers, lipid and extracellular vesicles, have not only upgraded the transfer of chemotherapeutics but also been extended to other treatments such as immunotherapy, gene therapy, phototherapy and theranostic approaches. These systems provide more accurate and multifunctional treatment tactics representing a way forward toward personalized oncology. Despite achieving great success in preclinical study, clinical translation is confronted with various difficulties in biology, tumor heterogeneity, safety, scaling up, and regulatory issues. The future of cancer nanovaccineis anticipated to grow centered towards the growth of intelligent, personalized and multifunctional therapeutic systems based on nanotechnology. This will envisage development of precise nanomedicine driven by biomarker, patient-specific or exosome-based drug delivery and advanced nanoplatforms with capacity of co-delivering genes, drugs and immunotherapy components. Also, “smart” nanocarriers that are responsive to stimuli and tumor-specific conditions such as pH, enzymes or temperature are likely to improve precise treatment and minimizing off-target toxicity. Concurrent with that, combination of nanotechnology with artificial intelligence (AI), machine learning and digital health systems such as the Internet of Nano-Things (IoNT) may help in real-time monitoring, optimized drug design and prediction of the toxicity potential of newly developed nanomaterials. Moreover, the use of safe-by-design approach, implementation of nanotoxicology guidelines and advanced preclinical models will be important for regulatory approval and clinical translation. In general, the future of nanomedicine should focus on developing biomarker-guided and patient-specific nanomedicine, multifunctional and stimuli-responsive platforms, rigorous immunotoxicity study, clinically relevant models and successful generation of nanomedicine lies in striking an equilibrium amongst innovation, safety and feasibility enablingmore effective, less toxic and real time patient centered cancer treatment. The systematic and critical analysis of the future of nanomedicine through the relationship between nanocarrier design, tumor-specific delivery, intracellular transport, stimuli-sensitive drug release, combination treatment and clinical translation is what makes this review innovative instead of discussing individual nanocarrier classes or therapeutic modalities alone.

### 5.1 Nanotoxicology andimmunotoxicity: risk and opportunity

Interaction of nanoparticle and immune system represent both challenges and opportunities for therapeutics. On one side, undesired immunotoxic effects of nanomedicine such as inflammation, complement activation-related pseudoallergy (CARPA), cytokine release, hypersensitivity and immune-mediated clearance can hamper the safety and therapeutic effectiveness. On the other side, these interactions can be strategically adjusted to increase antitumor immune response. For instance, nanocarriers can be designed to deliver tumor antigens, adjuvants, or immune-modulating agents precisely to immune cells, strengthening vaccine responses and checkpoint blockade therapies. A key determinant of these biological responses is the protein corona formed when nanoparticles reacts with biological fluids, which modifies their biological identity and can influence complement activation, opsonization, macrophage recognition, ligand accessibility, cellular uptake, biodistribution, clearance and toxicity. Pre-determined or treatment-mediated anti-PEG and anti-carrier antibodies may further promote complement activation, hypersensitivity and, in some formulations, accelerated blood clearance (ABC) following repeated administration, although these effects are formulation- and patient-specific. After accumulating the nanoparticles in clearance organs such as the liver and spleen, organ specific toxicity can occur in pulmonary and bone-marrow exposure depending on particle size, composition, surface properties, administration route and degradation behaviour. Material-specific effects, including metal-ion release, oxidative stress, mitochondrial and membrane damage, genotoxicity and fibrosis, together with the persistence of poorly degradable materials also contribute to acute or chronic toxicity. These responses can further change with patient-specific factors such as age, sex, hepatic and renal function, immune status, underlying disease and previous exposure to PEG-containing materials. Therefore, future studies should focus on safe-by-design nanomedicine, rigorous nanotoxicological and immunotoxicity assessment, clinically relevant models and patient-specific safety evaluation, while maintaining an appropriate balance between minimizing undesired immunotoxicity and maximizing desired immune activation.

### 5.2 Realistic expectations and translational difficulties

Although nanomedicine is often regarded as a “revolutionary” strategy in cancer treatment, realistic expectations are needed for its clinical success. Many promising nanocarriers exhibitpromising results *in vitro* and in animal models but lack the same efficacy in humans due to differences in tumor biology, vascular structure and immune environment. Along with that, expensive production rate, complex formulation strategy, low scalability and strict regulatory requirements make the clinical implementation of nanovaccines more difficult. Hence, future development should aim generation of simple, reproducible, budget friendly and clinically relevant study models to merge the gap between laboratory research and actual-world implications.

### 5.3 Artificial intelligence (AI) in cancer treatment

As already highlighted, artificial intelligence (AI), as transformative technology,has proved to be immense significanceine precise detection of cancer, diagnosis, drug development and personalized therapy. Early detection is now made possible using AI-assisted imaging models, containing mammography, magnetic resonance imaging (MRI) and ultrasound while AI-driven pathology platforms enable biomarker identification and improve prognostic assessment. Several critical aspects such as tracking risk stratification, estimating treatment responses and fixing individualized therapeutic strategies are now feasible through AI-based analysis of complex clinical, genetic and molecular datasets (Shi et al., 2023). Another crucial area is drug delivery and nanomedicine where AI is useful in formulating the correct design of drug to maximize bioavailability, minimize side effects, its pharmacokinetic property and developing patient-specific therapies. Recent studies have demonstrated the practical usage of AI in improving nanocarrier performance. Supervised machine-learning models can predict formulation and critical characteristics such as particle size, polydispersity, surface charge, encapsulation efficiency, drug loading, release kinetics and stability, while models incorporating biological and physicochemical descriptors can be used to estimate toxicity, cellular uptake and biodistribution. QSPR/QSAR approaches establish quantitative relationships between nanoparticle or molecular descriptors and biological or toxicological responses, whereas molecular simulations provide mechanistic information on drug–carrier interactions, membrane transport, protein adsorption and molecular-level organization rather than relying solely on prediction. Bayesian optimization is particularly useful for selecting successive formulations under multi-objective limits, such as maximizing drug loading and minimizing particle size or toxicity, with fewer experimental iterations. In contrast, generative models are being examined to propose new carrier compositions, ligands or molecular architectures that satisfy predefined structural and functional requirements. Increased tumor accumulation and stability can be achieved using AI-optimized liposomal doxorubicin formulations, while machine learning-guided polymeric nanocarriers exhibited enhanced gene silencing efficiency with minimum systemic toxicity (Wolf et al., 2022). AI-assisted imaging tools such as MRI, positron emission tomography helps real-time observation of nanocarrier distribution, drug discharge and therapeutic response. AI can also be of help in the field of microsphere-based drug transport systems, polymer selection, encapsulation efficiency, release kinetics and targeting accuracy. AI-based models can also be used to examine protein-corona composition and nano–bio interactions. AI combined with physicochemical, pharmacokinetic and patient-specific omics data can help may support the selection of targeting ligands and patient stratification. The AI approach can optimize nanoparticle characteristics, ligand density, payload composition and release behavior based on patient-specific omics and clinical data. Besides, AI is capable of accelerating drug discovery and repurposing by simulating drug interactions, recognizing potential targets and reducing reliance compared to conventional approach (Ghatage and Sharma, 2025). A FRET-based platform combining graphene quantum dots and mesoporous silica nanoparticles which enabled ATP-responsive intracellular drug release with fluorescence feedback has been documented. The system enhances targeting specificity and lowering systemic toxicity (Zheng et al., 2015). Similarly, Al Faraj et al., 2014 synthesized drug-loaded magnetic single-walled carbon nanotubes functionalized with targeting antibodies. This system performed simultaneous tumor-specific drug delivery and real-time monitoring through diffusion-weighted MRI. These multifunctional systems highlight the potential of intelligent nanocarriers to support adaptive, personalized and more effective cancer treatment strategies. However, these applications need to be interpreted primarily as predictive or optimization tools rather than direct experimental evidence. Current AI studies in nanomedicine are limited due to few, inconsistently reported datasets, heterogeneous experimental protocols, publication and selection bias, limited representation of negative results, and the risk of data leakage between training and test sets. Moreover, several models lack independent external validation and can have domain shift when applied across different laboratories, nanocarrier platforms, biological models or patient populations. Prospective testing of AI-generated formulations or predictions remains limited, and therefore a high model performance or computational prediction should not be considered equivalent to an experimentally demonstrated therapeutic outcome.

Therefore, before AI-guided nanomedicine can be considered reliable, future studies must emphasize standardized and FAIR data reporting, independent external authentication, explicit applicability-domain and uncertainty assessment, prospective experimental testing and ultimately clinically relevant validation. Newer approaches like explainable AI, federated learning, hybrid computational models, quantum computing and Internet-of-Medical-Things technologies are anticipated to contribute extensively towards AI-mediated cancer therapy (Deshmukh and Yadav, 2025).
